# Cyanobacterial Toxins of the Laurentian Great Lakes, Their Toxicological Effects, and Numerical Limits in Drinking Water

**DOI:** 10.3390/md15060160

**Published:** 2017-06-02

**Authors:** Todd R. Miller, Lucas J. Beversdorf, Chelsea A. Weirich, Sarah L. Bartlett

**Affiliations:** Joseph J. Zilber School of Public Health, University of Wisconsin-Milwaukee, Milwaukee, WI 53211, USA; lucasb@uwm.edu (L.J.B.); cweirich@uwm.edu (C.A.W.); bartle34@uwm.edu (S.L.B.)

**Keywords:** microcystin, saxitoxin, cylindrospermopsin, anatoxin-a, anatoxin-a(S), cyanobacteria, phosphatase inhibitor, acetylcholinesterase, neurotoxicity, drinking water

## Abstract

Cyanobacteria are ubiquitous phototrophic bacteria that inhabit diverse environments across the planet. Seasonally, they dominate many eutrophic lakes impacted by excess nitrogen (N) and phosphorus (P) forming dense accumulations of biomass known as cyanobacterial harmful algal blooms or cyanoHABs. Their dominance in eutrophic lakes is attributed to a variety of unique adaptations including N and P concentrating mechanisms, N_2_ fixation, colony formation that inhibits predation, vertical movement via gas vesicles, and the production of toxic or otherwise bioactive molecules. While some of these molecules have been explored for their medicinal benefits, others are potent toxins harmful to humans, animals, and other wildlife known as cyanotoxins. In humans these cyanotoxins affect various tissues, including the liver, central and peripheral nervous system, kidneys, and reproductive organs among others. They induce acute effects at low doses in the parts-per-billion range and some are tumor promoters linked to chronic diseases such as liver and colorectal cancer. The occurrence of cyanoHABs and cyanotoxins in lakes presents challenges for maintaining safe recreational aquatic environments and the production of potable drinking water. CyanoHABs are a growing problem in the North American (Laurentian) Great Lakes basin. This review summarizes information on the occurrence of cyanoHABs in the Great Lakes, toxicological effects of cyanotoxins, and appropriate numerical limits on cyanotoxins in finished drinking water.

## 1. Introduction

In freshwater environments, cyanobacteria (Cyanophyceae) dominate many nutrient rich lakes producing large accumulations of algal biomass seasonally during summer and autumn in temperate environments. The accumulation of excess algal biomass, or “algal blooms” in lakes and other aquatic environments impacts ecological and human health as well as the socioeconomic value of our water resources. While a natural occurrence, the eutrophication of water bodies and global climate change promote the frequency, duration, and magnitude of these blooms. Excess bloom material is consumed via respiration by heterotrophic microorganisms consuming oxygen (i.e., increased biological oxygen demand) leading to anoxic/hypoxic conditions detrimental to fish and other wildlife. In addition, decaying algal biomass produces noxious or unpleasant odors inhibiting recreational activities. Potent toxins and other constituents of some algae are harmful to aquatic organisms, and other animals including humans. The presence of these toxins and odors associated with algal blooms presents challenges for the use of freshwaters for producing high quality, aesthetically pleasing drinking water [1,2,3]. While many types of algae accumulate in aquatic environments, cyanobacteria are responsible for producing seasonal mass accumulations known as cyanobacterial harmful algal blooms or cyanoHABs. The focus of this review is on cyanoHAB occurrence and possible human health outcomes associated with cyanoHABs in the North American (Laurentian) Great Lakes (heretofore referred to as the Great Lakes), and proposed limits for human exposure in source and finished drinking water.

The ecology, toxicology, and physiology of cyanobacteria and cyanoHABs have been studied for more than a century providing rich information about their impacts on aquatic resources. Cyanobacteria (known colloquially as “blue-green algae”) are a ubiquitous and diverse group of photosynthetic gram-negative bacteria that inhabit both terrestrial and aquatic habitats throughout the planet. These bacteria were responsible for the oxygenation of early earth more than 3 billion years ago and are the precursors to chloroplast organelles in Eukaryotic algae and higher plants [4,5]. They are among the most important, diverse, and abundant photosynthetic organisms on the planet with lifestyles that range from essential symbionts of lichens and plants to the most abundant phototrophs in the world’s oceans (i.e., *Prochlorococcus*). 

An increasingly recognized characteristic of cyanobacteria is their ability to produce toxic or otherwise bioactive compounds that affect animal and human physiology. In most cases, these secondary metabolites are not necessary for normal functioning of the cell, but presumably provide some largely unknown benefit to the organism. The full diversity of these metabolites produced in nature is not known. Genomic studies suggest some cyanobacteria are capable of producing hundreds of different bioactive molecules with varying degrees of toxicity [6,7]. While some such compounds are of interest to the pharmaceutical industry for their medical benefits, others are potent toxins harmful to a variety of organisms. The presence of these compounds in aquatic environments, particularly lakes, presents challenges for water quality management and drinking water production.

Some areas of the Great Lakes have experienced increasing and/or persistent rates of eutrophication leading to massive cyanoHABs. This is troubling since the Great Lakes are a major source of drinking water. In recent years at least two drinking water plants drawing from Lake Erie have failed to reduce cyanotoxins to below acceptable levels. Furthermore, some areas such as southern Green Bay and the Lower Fox River basin are currently listed as impaired for drinking water use under the 303(d) listing of the Clean Water Act due to a combination of both cyanoHABs and industrial pollution.

### 1.1. Cyanotoxins Overview

Cyanotoxins associated with cyanoHABs in lakes are generally divided into three groups: liver toxins, neurotoxins, and dermatoxins. However, some cyanotoxins may not fit into these categories or have properties of more than one category. An overview of toxin structure, general mechanism of toxicity, and sources is covered below. An in-depth review of their toxicology is covered in Section 4 of this review.

#### 1.1.1. “Liver” Toxins

**Figure 1:**
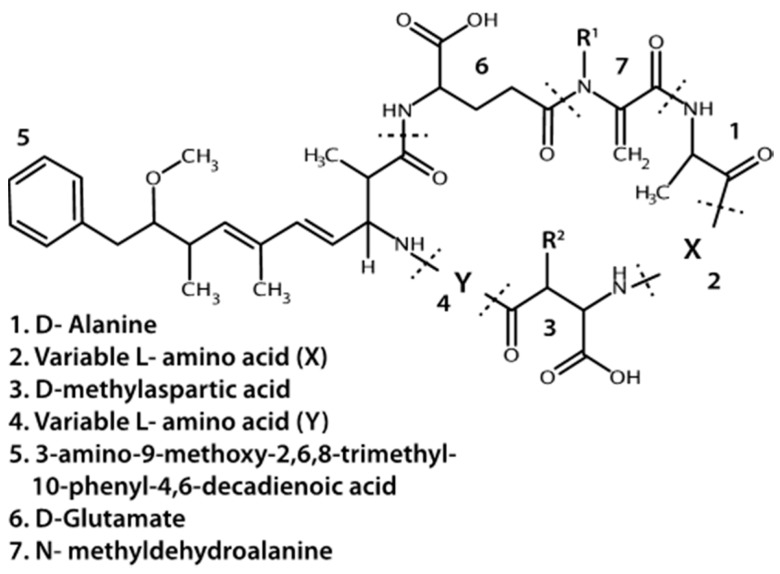
Base structure of microcystins. R^1^ and R^2^ may be a methyl group or hydrogen. Dotted lines indicate peptide bonds.

In the Great Lakes region, the most commonly observed or targeted cyanobacterial liver toxins are the microcystins (MCs) (Figure 1) [8,9,10,11]. They are cyclic heptapeptides with five non-protein amino acids and two variable protein amino acids. Methylations, hydroxylations, epimerizations and amino acid replacements lead to structural diversity of MCs where over a hundred different variants have been detected in lakes or cell cultures [12,13]. The number of different combinations of amino acid substitutions and structural alterations suggests at least 300,000 different congeners are theoretically possible (personal communication C.O. Miles, Norwegian Veterinary Institute). MCs covalently bind to and inhibit protein phosphatases type 1 and 2A in Eukaryotic cells, though other proteins and enzymes may also be inhibited by MCs [14]. In nature, MC with leucine and arginine at the variable protein amino acid positions (MCLR) and MC with two arginine residues (MCRR) are often reported to be the dominant variants detected, or at least targeted in environmental studies [8,15,16]. *Microcystis* is the dominant MC producer, but *Planktothrix*, and/or *Anabaena* taxa are also commonly associated with MC production [17,18,19]. A variety of other genera have been found to produce MCs including *Oscillatoria*, *Nostoc*, and *Gloeotrichia* [20,21,22]. With the recent identification of MC synthetase genes, the diversity of MC producers in nature is only beginning to be realized [18,23].

**Figure 2:**
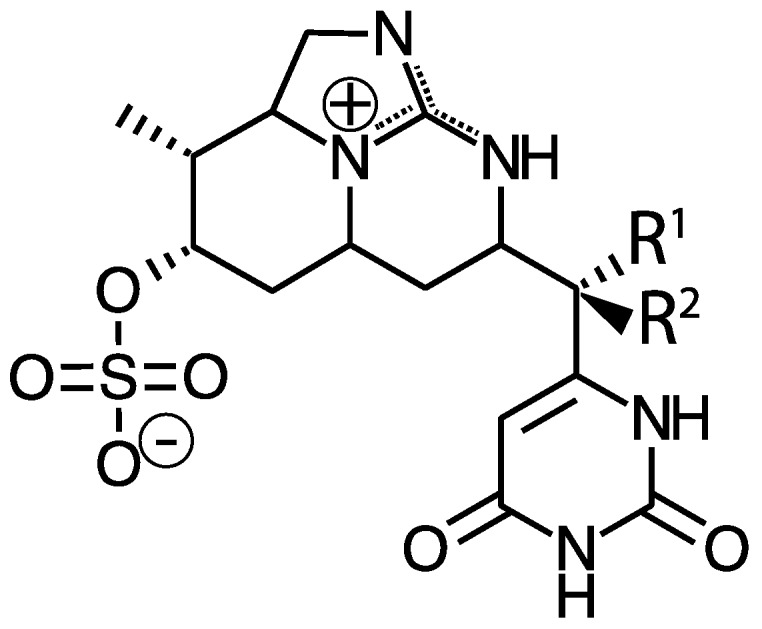
Structure of cylindrospermopsin (R^1^ = OH, R^2^ = H), 7-epicylindrospermopsin (R^1^ = H, R^2^ = OH), and 7-deoxycylindrospermopsin (R^1^ = H, R^2^ = H).

While MCs appear to be the most prevalent cyanotoxins, cyanobacteria also produce other liver toxins. Nodularin is a hepatotoxic cyclic pentapeptide with structural similarity to MCs. As such, it also inhibits protein phosphatases, but is primarily produced by *Nodularia spumigena* in brackish waters and may also occur in freshwaters [24]. Cylindrospermopsin and its analogs (e.g., 7-deoxycylindrospermopsin) is a sulfate ester of a tricyclic guanidine substituted with a hydroxymethyluracil (Figure 2). It is produced by *Cylindrospermopsis raciborskii*, but has also been identified in some other genera including *Aphanizomenon* as well as *Leptolyngbya* and *Phormidium*, dominated cyanobacterial mats in Antarctica [25]. It causes liver and kidney toxicity by inhibiting the synthesis of protein and glutathione with other toxic effects (see below and [26]). This compound also displays genotoxic effects in vitro and in vivo [27]. Although *C. raciborskii* is normally associated with (sub)tropical habitats, it is now considered an invasive species in temperate regions, including the Great Lakes [28,29].

#### 1.1.2. Neurotoxins

**Figure 3:**
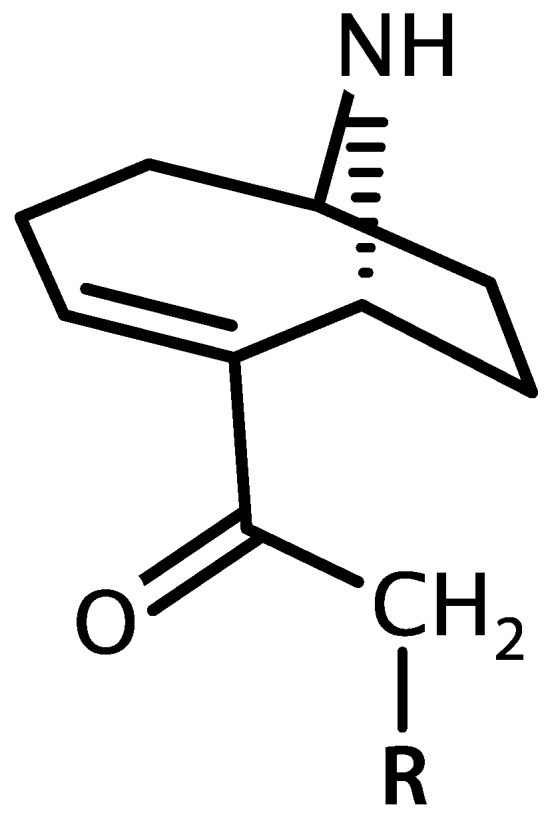
Structure of anatoxin-a (R = H) and homoanatoxin-a (R = CH_3_).

**Figure 4:**
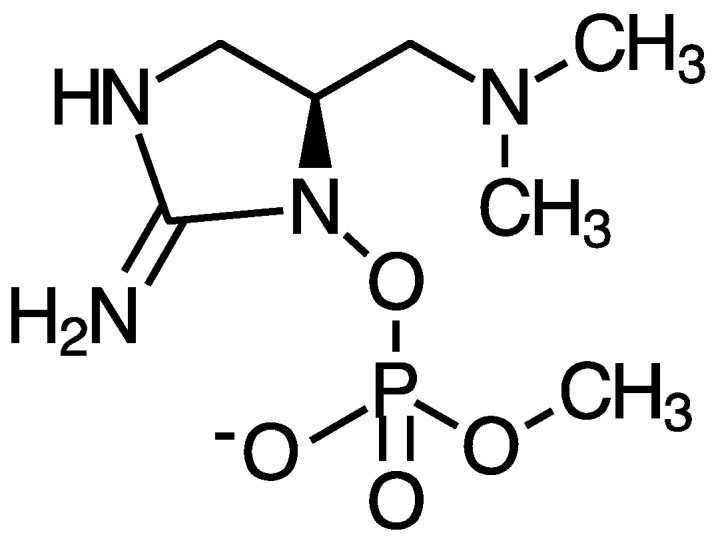
Structure of anatoxin-a(S).

Anatoxin-a and homoanatoxin-a are bicyclic alkaloids produced by species of *Anabaena*, *Oscillatoria* and *Aphanizomenon* among others (Figure 3) [30,31]. Anatoxin-a(S) is a naturally occurring organophosphate containing a methylphosphate ester attached to cyclic guanidine, structurally unrelated to anatoxin-a (Figure 4). Less is known about the distribution of anatoxin-a(S), but *Anabaena* species, particularly *Anabaena flos-aquae,* have been shown to produce this toxin [32]. Anatoxin-a and homoanatoxin-a are agonists of the nicotinic acetylcholine receptor and anatoxin-a(S) irreversibly binds to acetylcholinesterase in peripheral nerve cells [33,34,35,36,37]. The net effect of all anatoxins is uncontrolled activation of nicotinic and muscarinic acetylcholine receptors resulting in respiratory paralysis. 

Several other cyanobacterial neurotoxins are an emerging concern in North American recreational lakes. Recently, the neurotoxin beta-*N*-methylamino-l-alanine (BMAA) was found in diverse species of cyanobacteria [38]. It is normally associated with root symbionts, soil cyanobacteria (*Nostoc* species), of the Cycad tree and has been linked to amyotrophic lateral sclerosis/parkinsonism–dementia complex (ALS/PDC) in Guam and other human populations [39,40,41,42,43]. Thus, BMAA is associated with chronic illnesses with no evidence that it is acutely toxic. Cox et al. recently found that BMAA is also produced by every major order of cyanobacteria including common freshwater bloom forming species such as *Microcystis*, *Anabaena* and *Planktothrix* species [38]. Various other research groups have confirmed this finding [44], while others either failed to detect BMAA, found much lower concentrations, or detected isomers of BMAA instead [45,46,47]. Other reports indicate that BMAA and its isomers are present in some lakes and/or algal bloom material and dispersed in various organisms throughout the aquatic food web [43].

**Figure 5:**
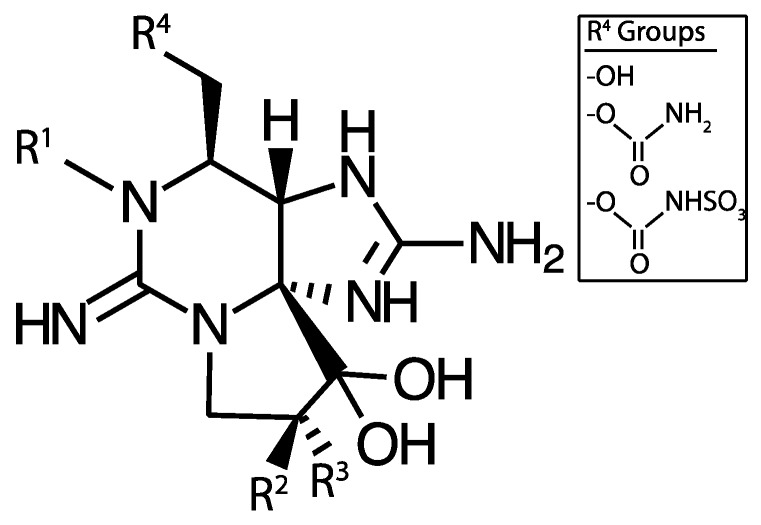
Saxitoxin base structure. R^1^, R^2^, and R^3^ may be a combination of H, OH, or OSO_3_^−^ and R^4^ may be one of OH, carbamoyl, or *N*-sulfo-carbamoyl groups.

Saxitoxin and its more than 50 analogs are tricyclic alkaloid neurotoxins that permanently block voltage-gated sodium channels in nerve cells causing paralysis (Figure 5). They are widely distributed in nature occurring in both fresh and saltwater and found in evolutionarily disparate organisms including cyanobacteria, dinoflagellates, and fish [48,49,50]. Historically this toxin is rarely found in northern temperate lakes, but the apparent spread of tropical or subtropical cyanobacterial species (i.e., *C. raciborskii*) capable of producing saxitoxin, into northern lakes including the Great Lakes suggests this toxin may become more prevalent [51]. In addition, genera common to temperate lakes (e.g., *Aphanizomenon*) have been found recently to produce saxitoxin in northern lakes [52]. Similarly, historically, *Lyngbya wollei* was associated with lakes in the Southwestern United States, but has now invaded some parts of the Great Lakes, especially the Western Basin of Lake Erie [53]. This organism blooms in thick mats at the sediment surface in shallow zones, and it produces two common saxitoxin variants as well as six that are unique to this species [54,55]. Saxitoxin has also been found in cyanobacterial benthic mat samples in the Arctic [56]. 

#### 1.1.3. Dermatoxins 

Various kinds of cutaneous skin rash and contact dermatitis are a common symptom reported anecdotally in cases of human exposure to cyanoHABs [57]. Cyanobacteria share many of the same structural features as other gram-negative bacteria (e.g., *Escherichia coli*) including the production of the irritant, lipopolysaccharide (LPS) located in the outer membrane. This antigenic substance recruits an immune response resulting in inflammation and exacerbates pathogenicity, including atopic dermatitis. It has been suggested that the LPS of cyanobacteria may cause or contribute to human illness, particularly in causing epidermal allergic reactions. However, there is little evidence in the scientific literature that skin contact with the LPS of cyanobacteria causes a skin rash [58,59]. Rather, it may be that skin rashes after cyanoHAB exposure are caused by one of hundreds of other bioactive metabolites produced by cyanobacteria. For example, cylindrospermopsin was shown to cause hypersensitivity reactions in the mouse ear-swelling test [60]. In addition, the marine cyanobacterium *Lyngbya mujuscula* produces tumor promoters and skin irritants including the indole alkaloid lyngbyatoxin A, and the polyacetates, aplysiatoxin and debromoaplysiatoxin (as reviewed by [61]). These molecules bind to phorbol ester receptors activating protein kinase C inducing excess phosphorylation of cellular proteins leading to disruption in cell cycle regulation and tumor formation with the initial symptoms being acute skin lesions [62]. Repeat exposure to these molecules is thought to cause skin cancer [63]. While *L. majuscula* does not occur in freshwater lakes, anecdotal evidence suggests that exposure to the freshwater species, *L. wollei* in freshwaters may induce dermatitis [64,65]. Whether *L. wollei* produces compounds similar to lyngbyatoxin A and the aplysiatoxins is unknown. 

#### 1.1.4. Miscellaneous

In addition to the potent toxins discussed above, cyanobacteria also produce various classes of linear and circular peptides that have potentially beneficial activities in mammalian cells. These include antivirals, antimicrobials, cytotoxic or anti-cancer compounds, and anti-fouling agents among others [66]. Many act by inhibiting phosphatases and proteases. For example, microginins produced by strains of *Microcystis* inhibit angiotension-converting enzyme, a protease, thereby preventing increases in blood pressure [67]. Anabaenopeptins are circular peptides that inhibit various human proteases and protein phosphatases PP1/2A [68,69]. Cyanopeptolins are serine protease inhibitors that may be useful in treating viral infections and asthma [70]. Like MCs, these bioactive peptides are synthesized non-ribosomally, or by a combination of non-ribosomal and polyketide synthetic pathways. They are modified in various ways producing a large amount of structural diversity. Most have been labeled as non-toxic, particularly at concentrations found in nature. However, exposure to these compounds in recreational environments and drinking water may prove to be problematic for ecological and human health since the full effect of cyanobacterial peptides individually, or in mixtures, is still largely unknown. For example, it was recently discovered that cyanopeptolin-1020 displays neurotoxicity in a zebrafish model and inhibits human kallikrein and trypsin in the low pico-to nanomolar range [71,72]. The 50% lethal concentration values against fairy shrimp (*Thamnocephalus platyurus*) for cyanopeptolin-1020 were similar to that of MCLR. Thus, cyanopeptolin, which was previously considered “non-toxic,” is now an emerging potent cyanobacterial toxin of interest. Therefore, the number of cyanotoxins present, including those just being discovered, complicates current strategies for properly monitoring and managing cyanotoxicity, and efforts focused solely on MCs may be inadequate to protect public health.

### 1.2. Characteristics of Bloom Forming Cyanobacteria

**Figure 6:**
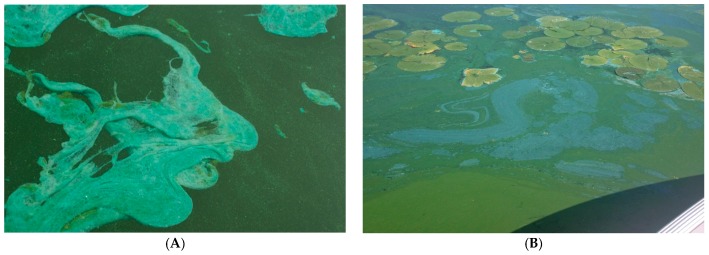
Cyanobacterial floating scums in (**A**) Lake Winnebago, WI in August 2013, and (**B**) Lake Mendota, WI in September 2008, showing the bright blue appearance due to C-phycocyanin.

Over their long history on this planet, cyanobacteria have evolved adaptations that favor their dominance in eutrophic lakes seasonally. Perhaps the most noticeable characteristic of many cyanoHAB species is their ability to multiply to high densities forming macroscopic colonies or groups of cells covered in a mucous polysaccharide sheath. In some cases, these colonies may coalesce into free-floating mats or “scums” on the surface of the lake with a bright blue or blue-green appearance due to the presence of C-phycocyanin, their major light harvesting pigment (Figure 6). Colony size affects cyanobacterial vertical movement, and aids in diffusion of nutrients and signaling molecules between cells [73,74]. Large colony size resists predation by zooplankton (unlike other non-colonial algae) and filter feeding organisms including *Dreissenids* (e.g., zebra mussels) [75,76]. The colonization of some lakes with *Dreissenids* has resulted in shifts in phytoplankton community composition to cyanobacterial dominance, such as in Lake Erie [10,77].

#### 1.2.1. Buoyancy

Nearly all cyanoHAB species possess protein gas vesicles that provide buoyancy and thus drive vertical movement of cells through the water column. These vesicles are cylindrical shaped structures formed by two hydrophobic proteins that diffusively accept gas and repel water [78,79]. With enough gas trapped in the vesicles, the cyanobacteria float upward toward the surface, where there is more photosynthetically active radiation. Photosynthesis produces carbohydrates, which may act as ballast, or increase cellular turgor pressure [80,81]. Depending upon the width of the gas vesicle, this pressure may result in the irreversible collapse of the gas vesicle leading to a loss of buoyancy and sinking. As such, buoyancy or vertical movement in cyanobacteria is regulated by gene expression of vesicle proteins, width of the cylindrical gas vesicle, photosynthesis, metabolism of carbohydrate ballast, and sunlight.

It has been proposed that vertical movement of cyanobacteria is an adaptation that allows cyanobacteria to capture nutrients in dark deeper layers of the lake, then float to the sunlit surface waters where photosynthesis and carbon fixation takes place [82]. This would give them a competitive advantage over other Eukaryotic phototrophs since long-term thermal stratification sequesters nitrogen (N) and phosphorus (P) in deeper waters at or below the thermocline while the nutrient-poor photic zone is generally limited to the upper few meters in most eutrophic lakes during the cyanobacterial growth season [83,84]. Thus, cyanobacteria may have a competitive advantage by overcoming this separation in nutrients and sunlight through vertical movement driven by gas vesicles. However, it is not clear if cyanobacteria vertically migrate to such lower depths where nutrients are sequestered, especially in deep lakes where the thermocline depth is often far below the photic zone [85]. A more important advantage for vertical movement may be in obtaining optimal light conditions and avoiding ultraviolet radiation and oxidative stress. CyanoHAB species have low light requirements, but can adapt to high or low light by varying relative amounts of chlorophyll-a and their major light harvesting pigment C-phycocyanin [80,86]. As such, some cyanobacteria are able to practice “self-shading,” producing blooms at the surface that shade out other phototrophic competitors while also growing at a depth where light may be limited [87,88].

#### 1.2.2. Nutrient acquisition

Cyanobacteria have a variety of mechanisms to compete for nutrients. Some cyanobacteria can fix atmospheric N providing a source of N when all other forms are scarce, particularly ammonium [89,90,91]. In most cases, N_2_ fixation has been shown to benefit both N fixing and non-N fixing cyanobacteria, likely due to secretion of fixed organic N from N fixing cells [81,86,87,92]. N_2_ fixation may explain why cyanobacterial dominance occurs in lakes with moderately low total N to total P ratios (<29:1) [87,93]. However, the timing of N_2_ fixation events may be more important in stimulating and sustaining blooms of toxic cyanobacteria [91]. Cyanobacteria also compete well for low levels of P and/or have lower P requirements compared to other phototrophs [94].

Most cyanobacteria also possess carbon, N, and P storage mechanisms. The carbon concentrating mechanism consists of protein transporters that concentrate bicarbonate within the cell to be used by RuBisCO (ribulose 1,5–bisphosphate carboxylase oxygenase) for carbon fixation [95]. Fixed carbon and energy may then be stored in glycogen and polyhydroxyalkanoate [96]. Similarly, in many bacteria including cyanobacteria, N and P are stored in cyanophycin and polyphosphate granules, respectively, thereby providing a source of N and P at a later time [97,98]. 

#### 1.2.3. Seasonality

The extent that these adaptations are expressed by cyanobacteria in nature favoring their dominance and resulting in cyanoHAB events depends on a variety of interacting physical, chemical, and biological factors that are only partially understood. Growth of cyanobacteria in nature is highly dependent on seasonal factors including water temperature, sunlight, and lake mixing. In north temperate environments, the optimal growth temperature for all cyanoHAB species is >15 °C, and the most prevalent toxic cyanoHAB species (e.g., *Microcystis*) have optimal growth temperatures >25 °C [99]. As such, cyanoHAB events generally begin when water temperature is highest and may persist as water temperatures slowly decline (i.e., in autumn). For example, in Lake Erie water temperature is not conducive for cyanoHABs until June with peak biomass between July and October [100]. Similar observations have been made for smaller lakes in the Great Lakes region including Lake Mendota, WI and Lake Winnebago, WI [83,101].

Water temperature is only one factor driving the growth of cyanobacteria in lakes. Nutrient availability either from external sources or internal recycling promotes cyanobacterial growth. Since the majority of external nutrients occur in spring when water temperatures are not conducive for cyanobacterial growth, Spring nutrient runoff is not likely to be immediately consumed by cyanobacteria to produce cyanobacterial biomass. To a greater extent, cyanobacteria thrive off of internal recycled P from other organisms or sediment as well as N_2_ fixation. Nutrient speciation is also important (i.e., organic or inorganic, N versus P) for the metabolic needs of the cyanobacteria at the time nutrients become available. For example, lack of N promotes N_2_ fixation by diazotrophic cyanobacteria, but this is more associated with the lack of ammonia than other N sources [102]. As such, N_2_ fixation occurs in the presence of nitrate [91,103,104]. Despite the association with eutrophic environments, on a temporal scale, cyanoHABs generally occur during periods when the standing stock of N and P are at their lowest [83,105]. Interestingly, some cyanobacteria (e.g., Planktothrix) have been shown to form blooms in some low nutrient lakes [106,107,108]. However, risk of toxic blooms greatly increases with rates of eutrophication [109]. 

#### 1.2.4. Physical Forces Causing cyanoHABs 

Besides growth of new biomass, cyanoHAB events can also occur due to vertical movement (floating/sinking) of cyanobacteria, wind and/or currents pushing floating cells, processes that do not necessarily require growth. Currents and prevailing winds can cause biomass to accumulate, pool, or pile up along shorelines creating bloom conditions [110]. Cells distributed throughout the water column can synchronize vertical movements thereby accumulating (i.e., bloom) simultaneously at the surface, often in the evening and on diurnal cycles [81]. Predicting how these factors interact with elicitors of cyanobacterial growth (e.g., nutrients and temperature) at temporal and spatial scales is a rich area of study in cyanoHAB modeling efforts [111].

#### 1.2.5. Species Dependent Effects

It is important to recognize that genus and/or strain specific differences among cyanobacteria present challenges in making gross generalizations about environmental drivers of cyanoHABs. Optimal growth temperature, toxin production, predator avoidance, colony size, shape, and density are just a few of the characteristics that have been shown to vary by species, strain, and genotype [99,112,113,114,115,116]. 

The rate of lake mixing and/or oscillations between mixing and thermal stratification are important factors determining the occurrence of cyanoHABs [87,117]. *Oscillatoriales* and other filamentous cyanobacteria are often associated with shallow turbid lakes that are rarely if ever thermally stratified [88]. These cyanobacteria are shade tolerant allowing them to outcompete other phototrophs in low light, turbid conditions. Reynolds et al. [118] demonstrated that cell shape, size, and density are principal factors in determining buoyancy of cyanobacteria or resistance to sinking and dispersal during lake mixing events. *Oscillatoria* compared to *Microcystis* and *Anabaena* have the lowest ballast per unit cell volume making them more resistant to sinking and dispersal during frequent mixing events that occur in shallow polymictic lakes. In contrast, blooms of spherical colonial species including *Microcystis* (such as occur in Lake Erie) are associated with infrequent mixing, thermally stratified conditions, which is likely due in large part to their relatively high ballast to cell volume ratio in both high and low light conditions. For example, cyanoHABs of *Microcystis* in Lake Erie and other lakes (e.g., Lake Taihu) are associated with low wind stress and thermal stratification [100].

The large diversity of cyanobacteria in nature prevents gross generalizations about their physiology and ecology. Efforts to model their responses to environmental variables at time scales relevant to human health (e.g., weekly if not hourly) are often site specific, largely non-transferrable between lakes. This may be in part due to a lack of sufficient data on a large number of lakes, or having the correct measurements. More research is needed in this regard.

## 2. Distribution of cyanoHABs in the Great Lakes Region

The five Great Lakes vary greatly in area and depth as well as trophic status. Green Bay alone ranges from hypereutrophic in the south to oligotrophic in the north. In addition, many embayments and tributaries or connecting water bodies exist that are more likely to support cyanoHABs, which could be transported into open water (e.g., Fox River to Green Bay and Maumee River to Lake Erie) [119]. 

**Figure 7:**
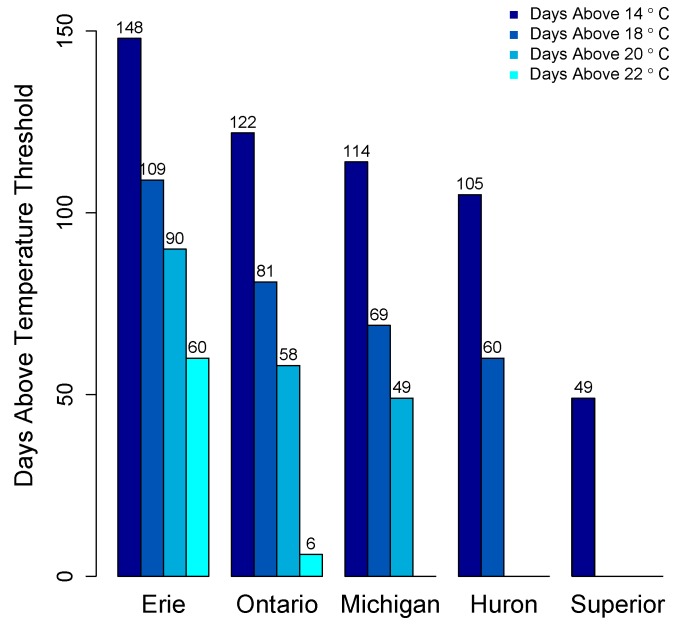
Average number of days surface water temperature above thresholds in the Great Lakes, 1992–2013. Data from National Oceanic and Atmospheric Administration, Great Lakes Environmental Research Laboratory, Great Lakes Sea Surface Environmental Analysis [1].

There is no systematic regular monitoring program for cyanotoxins in the Great Lakes. As such, the majority of data on cyanotoxin distribution comes from select peer reviewed studies, primarily in Lake Erie and non-peer reviewed data from governmental agencies. Appreciable growth of cyanoHABs is foremost dictated by water temperature. CyanoHABs rarely form in areas that do not have sustained water temperatures above at least 20 °C, though most cyanoHAB species likely form blooms at temperatures less than their optimal growth temperature. The average daily surface water temperatures for the past 21 years (1992–2013) in the Great Lakes show that Lakes Erie, Ontario, and Michigan exceed 20 °C for 49–90 days (Figure 7).

**Figure 8:**
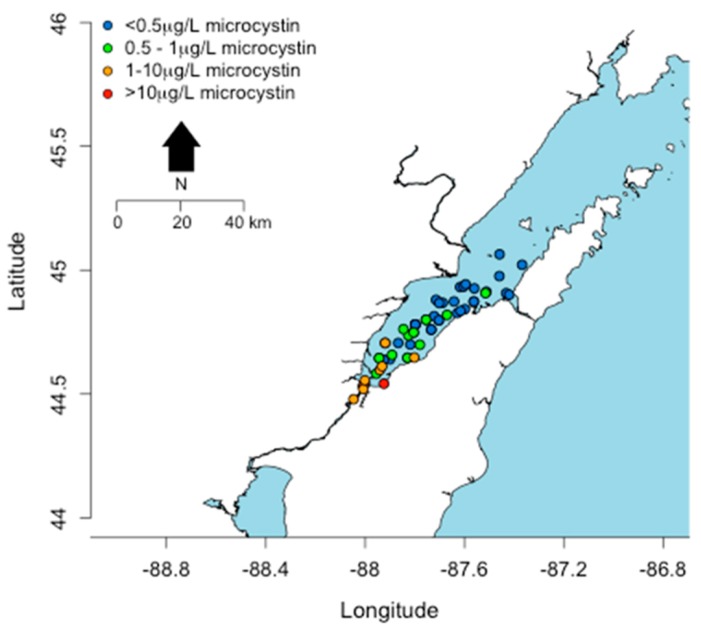
Concentrations (µg/L) of microcystins detected in a transect across Green Bay in August, 2014.

Only Lake Erie experiences surface water temperatures above 22 °C for any appreciable period of time. In other Great Lakes, certain embayments and near shore habitats reach temperatures well above 20 °C and sustain these temperatures into August. This includes Green Bay, Sandusky Bay, Grand Traverse Bay, Saginaw Bay, and other near-shore habitats particularly in Southern Lake Michigan. Connecting water bodies to Great Lakes may also contribute biomass and/or toxins including Lake Winnebago to Green Bay, Lake St. Clair to Lake Erie and Lake Macatawa to Lake Michigan among others. Cyanobacterial toxins have been detected in Lakes Erie, Ontario, and in certain areas of Lake Michigan including Grand Traverse Bay and Little Traverse Bay [120,121]. Green Bay is a shallow eutrophic embayment where cyanoHABs have been previously reported [77]. However there have been no published studies on cyanotoxin levels in Green Bay. We recently measured microcystins in transects of Green Bay (Figure 8) [122]. The mean and max levels of MCRR (0.97 and 8.5 µg/L) and MCLR (0.47 and 6.6 µg/L) were just under recreational risk levels. However, this was only one time point and blooms and toxins can vary widely over temporal scales [83,123,124,125].

Of all the Great Lakes, the Western Basin of Lake Erie and its connecting water bodies (i.e., Lake St. Clair, Maumee River) experience some of the most extensive cyanoHABs [126]. Historically, a variety of cyanobacterial species have been responsible for cyanoHABs in Lake Erie including filamentous N fixing cyanobacteria such as *Anabaena* and *Aphanizomenon*, as well as the non-N fixing *Microcystis* [11]. Early studies suggested that filamentous forms were dominant at times in Lake Erie in the 1970’s and 1980’s [127]. One study of preserved and recently collected sediment from Lake Erie suggests that *Microcystis* has always been the dominant MC producer since the 1970’s [128], although it is unclear how well cyanobacterial cells remain intact in deep sediments over the course of decades. In any case, CyanoHABs of toxic *Microcystis* species have escalated such that it is now clearly the dominant taxa during the primary growth season in the Western Basin of Lake Erie. 

**Figure 9:**
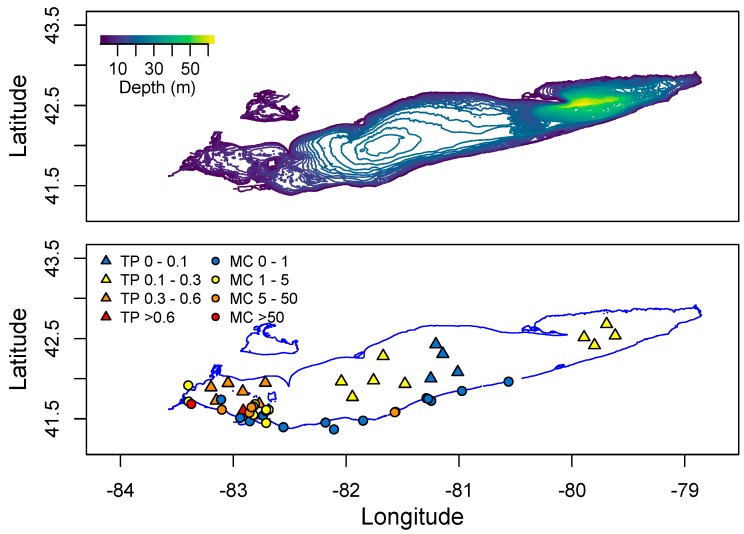
Distribution of total Phosphorus and microcystin across Lake Erie. Total P is the mean Spring concentration measured in 2008–2012. Microcystin data spans 2010–2015 from the Ohio EPA. No data is provided for Lake St. Clair north of Lake Erie.

One of the largest sources of nutrients to Lake Erie is the Maumee River, which drains a 16,835 square kilometer watershed, 80% of which is fertile agricultural land. This river enters the southwestern end of Lake Erie. Mapping Spring P levels across the lake, there is a clear gradient from low to high P in an East-West direction (Figure 9). Similarly, data from the Ohio Environmental Protection Agency [129] shows that the highest MC levels detected in the lake are found in the Western Basin (Figure 9).

This is the shallowest end of the lake allowing water temperatures to rise the fastest. Periodic mixing events resuspend nutrients while calm periods allow for the accumulation of biomass at the surface in sunlit waters. While this is a relatively shallow part of the lake, it is still deep enough to thermally stratify during summer months, which favors *Microcystis* dominance. Warming in deeper parts of the lake in the central and eastern basins is inhibited by cold deep water. As such cyanoHABs occur less frequently in these areas.

Since *Microcystis* is the dominant cyanobacteria in Lake Erie, the most commonly measured cyanotoxins are MCs. Surprisingly few peer-reviewed studies have published MC concentrations from Lake Erie or other Great Lakes. Rinta-Kanto et al. [9] measured MCs by protein phosphatase inhibition assay at various stations in the Lake Erie Western Basin at eight time points over three years and found maximum concentrations (14 µg/L) occurred in August. Millie et al. [130] measured intracellular MCs at sampling stations across the Western Basin of Lake Erie during single time points in 2003, 2004, and 2005. Intracellular MCs peaked at 0.13, 1.64, and 0.14 µg/L in 2003, 2004, and 2005, respectively. Dyble et al. [131] report a maximum of 4 and 0.38 µg/L intracellular and extracellular MCs, respectively in Western Lake Erie on a single cruise in 2004. One scum sample contained 58 µg/L intracellular MCs. Hu et al. [132] reported a maximum of approximately 2 µg/L MCs at a beach near the Ottawa National Wildlife Refuge on the Southwestern shore of Lake Erie in samples collected over a season (May–November) in 2012 with weekly sampling. In a survey of the Western Basin of Lake Erie, Wang et al. [133] detected MCLR in 16 of 36 samples in 2007. Concentrations reported in µg/g dry weight were highly variable from 0.5 to 3000 µg/g dry weight.

By comparison, recent data (non-peer reviewed data) reported by the Ohio EPA [129] spanning 2010–2015 shows similar or slightly higher concentrations of MCs at surface water locations in Lake Erie using the Enzyme Linked Immunosorbent Assay (ELISA). Table 1 shows mean and maximum MC levels reported at each site. Most locations are in the Western Basin except for sites 4, 13, 14, 18, 20, and 25, which are in the Middle and Eastern Basins. The overall mean and maximum MC concentrations at all surface water locations, combined is 126.9 and 3144 µg/L, respectively. This maximum of 3144 µg/L and the next highest data point at 570 µg/L are clear outliers in the dataset, though important indicators for extreme toxin loads that typically occur during peak bloom conditions or in wind-blown accumulations of biomass. Removing these outliers produces an average of 1.81 µg/L. Highest monthly max and mean MC concentrations occurred in July (34.49 and 3144 µg/L, respectively), followed by August (9.45 and 570 µg/L, respectively), and September (3.02 and 220 µg/L, respectively). With the outliers removed highest monthly averages occurred in August and September, each at ~3 µg/L.

Nine data points are reported for 2010 and 44 for 2011, otherwise, a similar number of data points are reported for 2012–2015 (62–77). The highest mean and maximum MC levels were over ten times greater in 2015 (52 µg/L) compared to 2014, 2013, and 2012 (4.00, 1.68, and 1.40 µg/L, respectively). Again, this result is driven by the two outliers. Removing these produces a mean MC level for 2015 (1.12 µg/L) similar to the previous three years. The Ohio EPA data suggest typical MC concentrations in the Western Basin of Lake Erie for the past several years of ~1–2 µg/L, with occasional extremes of one hundred to thousand fold higher concentrations, which typifies the dramatic spatiotemporal variability in cyanotoxin concentrations in lakes.

A recent study by the U.S. Geological Survey [134] measured MCs in Maumee Bay, Port Clinton, and Sandusky Bay (all in Lake Erie) in 2013 and 2014. Median levels were highest in Maumee Bay (6.8 µg/L), followed by Sandusky Bay (3.6 µg/L) and Port Clinton (0.4 µg/L). MC concentrations in Maumee Bay reached a maximum of 240 µg/L in late August of 2014 and were above 20 µg/L in five samples over a three-week period. In 2013 MCs were below 30 µg/L on all dates. 

## 3. Cyanotoxins in Drinking Water

The Great Lakes are currently used as a source of drinking water for over 35 million people. Lake Erie is the most affected by cyanotoxins and an estimated 11 million people rely on Lake Erie for drinking water. As such there is great interest in drinking water treatment strategies to remove cyanotoxins in this region. Other excellent reviews of cyanotoxin removal by drinking water treatment strategies have been published elsewhere [135,136]. Accordingly, this topic will not be covered here.

**Figure 10:**
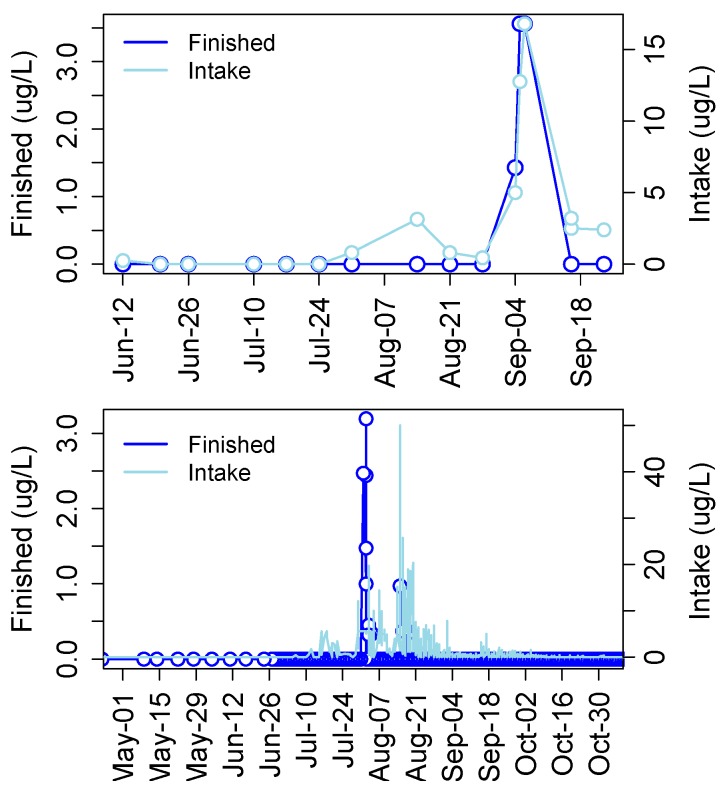
Concentrations of MCs in finished and intake drinking water at Carroll County (**top**) and Toledo (**bottom**), OH plants in 2013 and 2014, respectively.

Cyanotoxins have been detected in municipal drinking water in many developed and undeveloped or economically emerging countries including the United States, Canada, Argentina, Germany, China, Portugal, Spain, Poland, and Thailand among others (Table 2 in [137]). In a recent survey of finished drinking water supplies from 24 plants in the United States, 75% of samples tested positive for MCLR and some samples contained concentrations unacceptable for human consumption [138]. In 2013 MCs were detected in finished drinking water from the Carroll County Township drinking water facility in Ohio, which draws water from the Western Basin. Levels spiked to just over 3.5 μg/L in finished water coinciding with a large cyanoHAB event that produced over 15 μg/L MC in raw intake water (Figure 10). Then in 2014 MCs were detected in finished drinking water at the Toledo drinking water treatment plant at nearly 2.5 μg/L, 1.5 μg/L and 1 μg/L on three separate occasions that did not necessarily coincide with spikes in raw water (Figure 10).

The Ohio EPA has released data on concentrations of MCs in intake and finished drinking water from drinking water treatment plants (DWTPs) that draw water from Lake Erie and other locations in Ohio and neighboring states. Table 2 shows mean and maximum MC concentrations detected in intake water from Lake Erie by ELISA. The overall average and maximum level from all intakes is 1.04 and 340 µg/L, respectively. Nine DWTPs in the Ohio EPA dataset show detectable levels of MCs in finished drinking water (Table 3). Six of these draw water from Lake Erie and a seventh (Campbell Soup Factory) draws from the nearby Maumee River. Maximum levels in finished water at DWTPs drawing from Lake Erie range from 0.23 to above 3 µg/L, with max levels at the Carroll County and Toledo DWTPs. The Campbell Soup Factory had detects in finished water above 0.3 µg/L. Overall, the highest MC levels detected in finished drinking water are from the Celina DWTP. This plant draws water from Grand Lake St. Mary’s, which has produced cyanoHABs in recent years that have caused human illnesses, and which drains to the Maumee River and ultimately into Lake Erie.

These data suggest that current treatment strategies are not always effective at removing cyanotoxins and occasionally fail to reduce concentrations to below the World Health Organization’s (WHO) safe drinking water level of 1 μg/L MC. Thus there is a great need to fully understand toxicology of cyanotoxins (not just MCs), mechanisms of action, and reference dose levels producing harmful effects. Thus, the mechanisms of toxicity of the major cyanotoxins detected in the Great Lakes region are reviewed below within a historical context of their discovery.

## 4. Mechanisms of Toxicity

In 1878 George Francis, of Adelaide, South Australia published his observations of mysterious animal deaths (horses, sheep, dog, pig) after they drank from Lake Alexandrina in Milang [139]. He observed that animal deaths occurred when blooms of cyanobacteria, perhaps *Nodularia*, were blown by the wind toward the shore. Francis explained that the animals died within hours after drinking lake water exhibiting a range of symptoms consistent with neurotoxicity including stupor and unconsciousness, convulsions with head drawn back, and rigid spasm. Interestingly, he noted that the animals did not drink from “*puddles where scum has collected*”, but avoided those areas and drank from “fresh” waters. As such, he wrote, “… *the poisoning is not caused by drinking a putrescent fluid full of bacteria as at first supposed*.” These statements are widely cited as the first scientific reports implicating cyanobacteria in the production of one or more potent toxic substances. 

Following this report, others followed in the United States, Canada, and elsewhere during the 1880’s and early 1900’s as summarized by Fitch and colleagues in 1934 [140]. These reports confirmed Francis’ observations suggesting that poisonous compounds were associated with blooms of cyanobacteria. Early experiments with laboratory animals involved intraperitoneal injection (IP) of raw or crude extracts of bloom material, cultured cells, or crude extracts of both. Results from these early studies formed the recognition that cyanobacteria (or other organisms associated with them) were likely responsible for toxin production; however, procedures for cyanobacterial isolation/culturing and toxin purification were unknown until the 1970’s. Therefore, studies before this time are difficult to interpret and will only be considered briefly here. It should be noted that even current methods for growing cyanobacteria free of contaminating organisms (e.g., heterotrophic bacteria) remain difficult. Nonetheless, modern toxicological studies have at least used purified toxin material.

### 4.1. Commonly Found Cyanotoxins

The most frequently occurring and/or detected cyanotoxins in the Great Lakes region are MCs, anatoxins, cylindrospermopsins, and saxitoxins. Evidence for their mechanism of human and animal toxicity is discussed below.

#### 4.1.1. Microcystins

It had been observed by 1930 that various species of *Microcystis* were often the dominant organism present in lake water associated with animal deaths. Fitch et al. [140] were among the first to describe the effects of *Microcystis* bloom material on laboratory animals through IP injection. Guinea pigs, rabbits, and pigeons died rapidly (minutes to hours) producing similar symptoms including restlessness, urination, defecation, deep breathing, hind-quarter weakness, coughing, salivation, lachrymation, and clonic spasms. They observed that toxicity of bloom samples decreased when stored in a refrigerator, probably due to degradation of the toxins by associated heterotrophic bacteria. In addition, it is likely that multiple substances were acting on the animals to cause the illness since raw bloom material was used. Thus, efforts were made to isolate and culture *Microcystis aeruginosa* in the laboratory for toxicological studies [141,142].

In 1946 (just after the end of World War II), Ashworth and Mason [143] made detailed observations of pathological changes in rats after injection with a chloroform, acetone, ether extract of *M. aeruginosa* culture. This study as well as others (e.g., [144]) established that gross pathological changes associated with *M. aeruginosa* are primarily observed in the liver. This study also showed that the effects were similar to other hepatotoxic agents involving cytolysis of liver cells. They reported that after injection with a sub-lethal dose of *M. aeruginosa* culture, the liver goes through defined stages. Approximately 30 min after exposure the liver becomes slightly enlarged, red, and tense. At three to six hours, liver weight is 25% above the controls, there is increased blood content of parenchyma, and the tissue becomes soft and friable with all lobes affected. At 2–3 days, the liver has shrunk by 2/3, it is yellow and mottled, and blood coagulation is slow. At five days after the maximal sub-lethal dose, the liver returns to a normal state. This is the result of a one-time exposure to a high, but sub-lethal dose.

It was not until 1959 that a MC toxin (presumably) was partially purified and characterized from *M. aeruginosa*. This was largely enabled by the isolation and mass culturing of *M. aeruginosa* [142]. Bishop et al. [145] purified a toxic fraction (i.e., by mouse bioassay) from *M. aeruginosa* NRC-1 using Soxhlet extraction with methanol. Five peptides were detected upon electrophoresis of the crude extract at pH 7, and one was found to be acutely toxic in the mouse bioassay (IP LD_50_ = 460 µg/kg body weight (b.w.)). This peptide (or mixture thereof) was likely an MC, but the amino acid content given was not consistent with the MC base structure. This fraction was identified as the “fast death factor” earlier described by Hughes et al. [142]. It should be noted that Paul R. Gorham’s laboratory was behind much of the early work showing that cyanobacteria produce toxins harmful to a variety of organisms in controlled settings, as well as some of the initial characterizations of cyanotoxin properties.

In 1965 Konst et al. [146] conducted animal trials with guinea pig, rabbit, mice, duck, chicken, lamb, and calf. As the starting material, they used freeze-dried *M. aeruginosa* and administered it by oral or the IP route. Again the liver was found to be the most affected organ in both exposures with minor abnormalities in heart and lungs. A significant finding of this early study was that the oral route was much less toxic (40×) than IP route. In addition, as had been observed by others (e.g., Ashworth and Mason), blood coagulation was slow in dosed animals likely due to liver damage and depression of prothrombin levels. 

While these early studies did not use pure toxin, the analysis of whole cell toxicity is somewhat enlightening. For example, Konst et al. as well as Hughes et al. observed rapid death and supposed neurotoxic effects in mice injected with *M. aeruginosa* including convulsions, dragging of hind legs, and loss of equilibrium. Were these symptoms due to acute liver failure, neurotoxicity of MC, or other neurotoxins associated with *M. aeruginosa*? This was unknown at that time. It is important information as it corroborates recent findings that MC is a neurotoxin (see below), and that at least some strains of *M. aeruginosa* are capable of producing other non-ribosomally synthesized peptides that show neurotoxicity (e.g., cyanopeptolin) [71]. 

#### 4.1.2. Pathological Studies Using Pure MC Toxin

A description of the definitive structure of MC was given by Botes et al. [147] and Rhinehart et al. [148]. These studies and prior work established that *M. aeruginosa* does indeed produce cyclical toxic peptides as reported by Bishop et al. (1959). Structural elucidation paved the way for controlled toxicological studies using purified toxin, rather than bloom material or cultured cells potentially containing a mixture of toxins of unknown quantity. Culturing and toxin isolation studies were primarily performed by Gorham and Carmichael [149], which were critical to the elucidation of cyanotoxin properties. In addition, analytical methods were developed soon after to quantify the amount of toxin used [150], as well as appropriate extraction methods for MCs [151].

In 1989, Hooser et al. [152] performed one of the first studies of acute toxicity of MCLR (i.e., MC with leucine and arginine in the two variable amino acid positions) using purified toxin (by preparative high-pressure liquid chromatography) in rats and mice via IP exposure. The lowest one time dose producing death in rats and mice within 24 h and 90 min, respectively, was 160 µg/kg·b.w. in rats and 100 µg/kg·b.w. in mice by i.p injection. At IP doses of 80 and 40 µg/kg·b.w., male and female rats, respectively, had no clinical signs of toxicity, or gross or microscopic lesions. At 120 and 80 µg/kg·b.w. a portion of male and female rats, respectively, showed clinical signs of toxicity. All animals showing clinical signs eventually died. Similar results were found by Runnegar et al. [153] using both MCLR and MCYM where 84 µg/kg·b.w. resulted in nearly complete inhibition of liver protein phosphatase followed by death.

**Figure 11:**
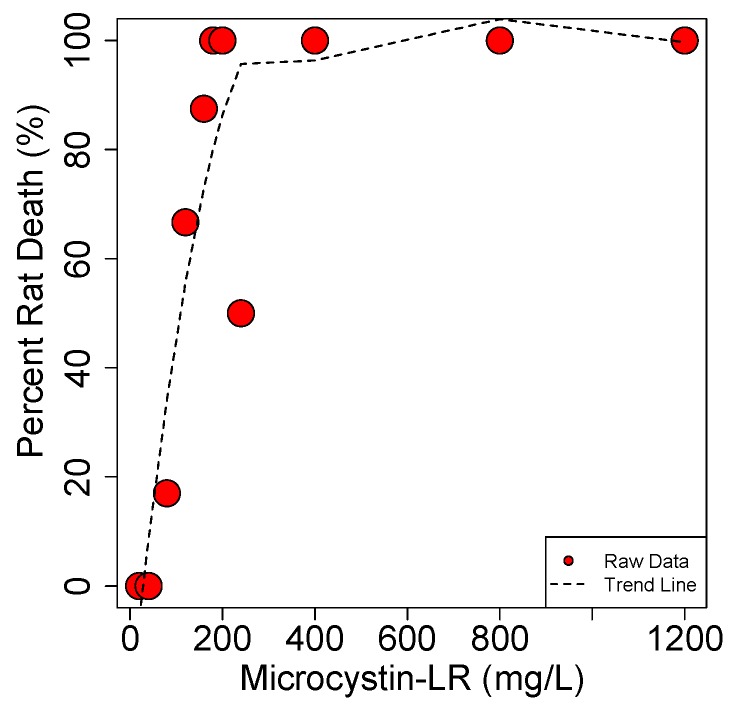
Dose-to-death curve for MCLR in the rat. Data from Hooser et al. 1989 [152].

These results show that MCs display an extremely steep dose to death curve (Figure 11). The study by Hooser et al. shows that the difference between a dose that causes death and complete recovery (with IP exposure) is less than a factor of 2. Similarly, Lovell et al. [154] showed that 25 µg/kg·b.w. is the max dose resulting in no death in mice while the LD_50_ is just 7.5 µg/kg·b.w. higher at 32.5 µg/kg·b.w.

In the Hooser et al. study, time to death with MCLR exposure via IP injection ranged from 20 to 32 h (120–240 µg/kg·b.w.) or 6 to 8 h (400–1200 µg/kg·b.w.) and symptoms were largely lethargy and ruffling of fur. Liver weight was significantly higher for all dosage levels. Alanine aminotransferase, alkaline phosphatase, urea, and creatine serum levels increased over time in all treated animals suggesting liver damage at all dosages. There was also a decrease in serum glucose levels. Upon pathological examination, it was found that a breakdown in sinusoidal liver endothelial cells and hepatocyte dissociation resulted in the presence of hepatocytes and cellular debris in the pulmonary artery and lungs. Hepatocyte damage preceded the presence of these cells in lungs thus disproving the earlier theory that liver damage is due to pulmonary thrombosis. 

#### 4.1.3. Repeat MC Oral Dose Studies

By comparison, MCs are 30–100 fold less toxic by oral exposure in rats and mice. Fawell et al. [155] conducted tests of MCLR (pure toxin from Calbiochem-Novabiochem) toxicity in mouse and rat through both IP injection and oral gavage. Experiments were carried out to examine acute, developmental, and long-term (13 weeks) effects. As in previous studies, symptoms of acute exposure included convulsions, hypoactivity, prostration, and slow respiration. Death by oral route in mice occurred with a single dose of 1580 µg/kg·b.w. while no death occurred at 500 µg/kg·b.w. The authors concluded a no observed adverse effect level (NOAEL) in the mouse through oral doses of 40 µg/kg·b.w. per day over the 13-week period. Criteria for the effect level included gross and microscopic liver pathology and blood chemistry. At the next highest level of 200 µg/kg·b.w. per day, slight changes in blood chemistry were noted, but there was some uncertainty about these results. Fetal development was assessed, but the mice were not followed after birth (i.e., for learning, memory, or overall cognitive deficits). Interspecies differences were noted as MCLR was somewhat less toxic to rats. The results of this study were similar to those of Falconer et al. [156] using pigs in an oral exposure route study. These two studies are currently the basis for the WHO maximum allowable concentration for MCLR in drinking water (1 µg/L) that exists today 16 years later. 

R. Heinze [157] also performed a repeat oral dose study of MC toxicity in rats. This study provided male adult rats MCLR in drinking water for 28 days at dosages of 50, or 150 µg/(kg·b.w.·day). Body weights were measured weekly. After the exposure, endpoints were determined including body and organ weights, liver serum enzyme activity, blood cell counts, differential counts of leucocytes, hematocrit and hemoglobin, and histopathology of liver and kidney specimens. At both dosage levels liver weight was increased as was serum liver enzymes alkaline phosphatase and lactate dehydrogenase. In addition, histopathology showed evidence of liver damage or lesions in both groups. Thus, the lowest dose showing toxicity by these endpoints was 50 µg/(kg·day).

#### 4.1.4. Effects on Other Tissues from Oral Exposure to MCs

In addition to liver damage MCs have also been shown to affect brain and reproductive tissues [158,159,160,161,162,163,164,165,166,167]. Of note for risk analysis are those studies that examine effects from repeat oral dose exposures. In a recent study, Li et al. [168] exposed rats orally to MCLR (0.2, 1.0, and 5.0 µg/kg·b.w.) by intragastric gavage for 8 weeks every two days and measured liver serum enzymes and effects on neurobehavior, or learning using the Morris water maze test. This test measures how quickly the animals learn to find a platform in a circular pool to escape the water. At 5 µg/kg·b.w. there was a significant increase in serum cholinesterase levels and escape latency in the water maze test after the 8 weeks of oral exposure to MCLR. Furthermore, post-mortem analysis demonstrated accumulation of nitric oxide and nitric oxide producing cells in regions of the hippocampus. Nitric oxide acts as a neurotransmitter in the brain and is associated with learning and memory (reviewed in [169]). Based on this study, central nervous system toxicity of MCLR begins to occur at 5 µg/kg·b.w. delivered every 2 days.

Li et al. [170] examined the neuro-developmental effects of MCLR in a repeat maternal oral dose study. In this study Sprague-Dawley female rats (28 days old) were exposed to 1.0, 5.0, or 20 µg/kg·b.w. MCLR by gavage every 48 h for 8 weeks. After the 8 weeks the mice were mated and the offspring examined for adverse effects including body weight, morphological aberrations (external malformations, hair appearance, incisor eruption, and bilateral eye opening), deficiencies in motor development, learning and memory delays (i.e., Morris water maze test), histopathological analysis of hippocampus CA1 regions, and lipid peroxidation and antioxidant indices in the hippocampus. Cliff avoidance time decreased in pups seven days postnatal at all exposure levels. In addition, performance in the Morris water maze test at postnatal day 60 was diminished. Specifically, the frequency of entering the platform of all exposed male offspring and female offspring from the 5 and 20 µg/L exposed group was significantly lower compared to controls. In addition, swimming speed of female offspring from mothers treated with 20 µg/kg·b.w. MCLR was significantly decreased. Malondialdehyde contents and superoxide dismutase activity were significantly higher in the highest exposure groups.

Effects of MCLR on reproductive tissues have also been reported in repeat dose oral exposure studies. Using mice, Chen et al. [171] examined the effects of oral exposure to MCLR in drinking water on sperm count and motility, body and testis weights, serum testosterone, and apoptosis in testicular tissue. For the exposure, male mice were given sterile water *ad libitum* containing MCLR at 1, 3.2, and 10 µg/L for three or six months. At 3.2 and 10 µg/L levels sperm counts and motility were significantly decreased after both three and six months. In addition, serum testosterone and luteinizing hormone levels were decreased in the 3.2 and 10 µg/L exposure levels after 3 and 6 months. At 6 months there was a clear dose response relationship between exposure level and apoptosis of testicular cells.

The reproductive toxicity of MCLR reported by Chen et al. is corroborated by a variety of other in vivo studies using IP injection and in vitro studies using cultured cells or isolated reproductive tissue. Ding et al. exposed male eight-week old mice to 3.3–6.7 µg/kg·b.w. MCLR in cell extracts daily for 14 days by IP injection and then measured toxic effects on reproductive organs. There was a significant decrease in body weight, sperm viability, rapid sperm motility, and an increase in percent sperm immobility. Li et al. (2008) observed that in mice exposed to 5 µg MCLR/kg·b.w. /day via IP injection for 28 days sperm motility significantly decreased while at 15 µg/kg·b.w./ day there was a decrease in testis weight, sperm concentration, serum testosterone, human luteinizing hormone, and follicle stimulating hormone. In other studies chromatin condensation, nuclei fragmentation and DNA fragmentation in testes cells has also been reported as a result of exposure to MCLR [162]. Increases in p53, Bax, and caspases in testis tissue have also been observed as signs of programmed cell death [162,172,173].

#### 4.1.5. Molecular Mechanism of MC Toxicity

These important studies established criteria for protecting human populations from injury due to acute MC exposure. They establish that the acute and overall observable pathological effects after consumption of water containing MCs occurs at relatively high concentrations >1000 µg/kg·b.w. Dosages at these levels are unlikely to occur with treated drinking water with even minimal primary treatment. In recent years, the effects of chronic low-dose exposure to MCs as well as toxicity to tissues other than the liver have been examined. These studies are based on the known mode of action of MCs. The molecular mechanism of MC toxicity resembles that of other biomolecules. The list of naturally occurring molecules that inhibit phosphatases in nature includes dinophysistoxins, calyculin, dragmacidins, tautomycin, tautomycetin, cytostatins, phospholine, leustroducsins, phoslactomycins, fostriecin, cantharidin, okadaic acid as well as MCs [174]. In particular, okadaic acid is associated with chronic diseases such as tumor production and cancer. Okadaic acid is a marine biotoxin produced by dinoflagellates and it accumulates in various host tissues including shellfish and sponges [175,176,177]. It is a tumor promoter and potent inhibitor of PP1/2A. Yoshizawa et al. [178] discovered that in cytosolic fractions of mouse liver, MCLR inhibited the binding of okadaic acid to protein phosphatases, increasing protein phosphorylation and decreasing phosphatase activity in 50% of controls using nanomolar levels of MCLR. Structural studies show that the methylene carbon of the methyl-dehydroalanine residue of MCs covalently binds to a cysteine residue on the C subunit of PP1/2A phosphatases leading to enzyme inhibition [179]. This also has the effect of preventing the detection of MCs in exposed individuals by most approaches and accumulation of the bound product in host tissues, primarily the liver. Note, however, that recent studies suggest that MC bound to thiols might disassociate over time. Thus, it is possible that some portion of protein-bound MCs might still be considered potentially toxic.

The consequences of PP1/2A inhibition by MCs are of significance for both acute and chronic toxicity. The PP1/2A phosphatases are essential for cellular survival. They help maintain homeostasis by controlling the activity of signal transduction pathways through the dephosphorylation of effector molecules. They consist of three subunits (A, B, and C) where C is catalytic, A is structural, and B determines substrate specificity. Multiple types of each subunit are present in human cells providing functional diversity. Phosphatase subunits bind forming an active structure only when the C subunit is methylated at the terminal Leu at residue 309. PP2A is one of the most abundant proteins in Eukaryotic cells and the most common phosphatase. Along with other proteins, active PP2A down regulates pathways involved in instigating cell proliferation and growth, protein synthesis, and resistance to cell death or apoptosis (as reviewed by [180]). As such, it is a tumor suppressor and a new target for anti-cancer therapies [181,182]. Indeed, PP2A is mutated or altered in many types of cancer cells [183,184,185]. The activity of PP2A in response to stressors including DNA damage is controlled by its methylation state. Both MCLR and okadaic acid have been shown to directly inhibit the methylation of PP2A preventing the formation of an active PP2A holoenzyme. Thus, MCs and okadaic acid are tumor-promoters through kinase driven malignancy [182,186,187]. In 2010, MCLR was classified as being “possibly carcinogenic to humans (Group 2B)” by the International Agency for Research on Cancer [188]. 

This mechanism suggests a dualistic response to MCs by mammalian cells. At a high dose, MCs cause massive changes in cell morphology through cytoskeletal rearrangements and oxidative stress, leading to loss of cell-to-cell adhesion and cell death [189,190,191]. In a chronic model of low level exposure, constant mild PP2A inhibition leads to reprogramming of the cell, runaway cell growth, and tumor production, analogous to the effects of endogenous human protein CIP2A (cancerous inhibitor of PP2A) associated with breast and lung cancer [192,193,194,195]. The level of MC required to cause complete liver failure and death is known (>1000 µg/kg·b.w. via oral dose), but the level required over time in multiple exposures to cause cellular reprogramming of liver cells (or other tissues) and tumor growth is unknown [196].

MCs also inhibit PP1/2A in brain cells [197,198,199]. In the central nervous system, PP1/2A control long term potentiation and long-term depression through dephosphorylation of the AMPA (α-amino-3-hydroxy-5-methyl-4-isoxazolepropionic acid) receptor at glutamatergic synapses. These processes facilitate learning and memory. There is evidence that MCs migrate to brain tissues following exposure [200] and are able to cross the blood-brain barrier [201,202,203]. Inhibition of PP1/2A by MCs in rat brain has been shown to block long-term potentiation induction suggesting that MC exposure may induce cognitive delays [167]. Indeed, direct injection of MC to rat hippocampus at femtogram levels has been shown to affect learning and memory [165,166,170]. MCs have also been shown to alter fish behavior and learning as well as increase acetylcholinesterase activity in fish brain [164]. In the well-described human MC intoxication event of dialysis patients in Caruaru, Brazil, patients experienced symptoms of neurotoxicity including deafness, tinnitus, and intermittent blindness [204]. Thus, it appears that MCs are central nervous system toxins; however, there is currently a lack of information concerning effects of long-term chronic exposure to low levels of MC that may affect processes such as learning and memory, particularly in children whose development is dependent on these activities. Such issues were not addressed in developing the WHO 1 µg/L recommendation for drinking water.

Health outcomes and target organs affected by MCs are highly dependent on OATPs (organic anion transporting polypeptides) that transport MCs into Eukaryotic cells. There are 11 OATPs in human cells. OATPs 1A2, 1B1, and 1B3 have been shown to transport MCs [205]. OATP 1A2 is expressed in the blood-brain barrier [206,207,208,209], kidney [208], cholangiocytes (bile duct epithelial cells) [210,211], testes, and enterocytes [212]. OATP 1B1 and 1B3 are restricted to the liver under normal conditions. Thus, in addition to liver and brain, the kidney, bile duct, testes, and intestines are all additional potential targets of MC toxicity. Furthermore, other OATPs may be involved in MC transport. For example, OATPs 3A1 and 4A1 have been implicated in the uptake of MCs and these OATPs are distributed ubiquitously in human tissues [213,214]. 

OATPs play a large role in determining one’s sensitivity to xenobiotics, pharmaceuticals, and biotoxins, including MCs as well as other amphiphilic algal metabolites (e.g., peptides) [215]. Genetic variations in OATP genes can increase or decrease OATP protein transport activity resulting in altered pharmacokinetics [216,217,218,219]. For example, healthy individuals carrying the common thymidine to cytosine single-nucleotide polymorphism (SNP) at base pair 521 of the OATP 1B1 gene (that transports MCs) are more sensitive to statins and other important medications (and potentially MCs) [220]. OATP 1B1 alleles with this SNP have decreased transport activity toward some statins, significantly increasing statin serum concentrations. On the other hand, this allele has increased activity towards other drugs and toxins (as reviewed by [219]). Genetic alterations in OATPs are currently a robust field of inquiry. Over 200 SNPs have been identified in human OATP genes, which may be more common in Asian populations [221]. The rate of OATP 1B1 and 1B3 expression shows significant inter-individual variability and the expression levels of OATP 1B1 is correlated with SNPs in OATP 1B1 genes [222]. It is therefore likely that metabolism, clearance rate of MCs, and overall human health outcomes associated with MC exposure are highly dependent upon an individual’s genetics, particularly with respect to OATP transporters. In addition, it is not clear whether rodent and human OATPs share the same tissue distribution, activity/substrate interactions, and overall phenotypic response. Therefore, rodent studies of MC toxicity (discussed above) may not reflect true toxicity in humans. As such, the 1000-fold safety factor employed in calculating the WHO maximum concentration guideline value for drinking water is clearly warranted. 

### 4.2. Anatoxin-a

In early reports, blooms of other cyanobacteria, including *Anabaena flos-aquae* and *Aphanizomenon flos-aquae*, were implicated in the death of water foul and domesticated animals [140]. Extracts of both *Anabaena* and *Aphanizomenon* produced very fast deaths (within 7 min) through IP injection with symptoms of neurotoxic poisoning including convulsions, limb twitching, eventual paralysis, and death [141,223,224,225]. The previously defined “aphantoxins” from *Aph. flos-aquae* have now been determined to be saxitoxins and gonyautoxins. Carmichael et al. [225] determined that the toxin from *Ana*. *flos*-*aquae* strain NRC-44-1 (isolated from Burton Lake, Saskatchewan, SK, Canada) was a neuromuscular depolarizing agent and specifically a cholinergic agonist acting upon nicotinic acetycholine receptors with high affinity and muscarinic receptors with low affinity [32,226]. The toxin was named anatoxin-a or *Anabaena* toxin “A” to discriminate it from other toxins in *Anabaena* species. The toxin structure was proposed by O. E. Edwards to C. S. Huber in personal communications and the crystal structure was determined by Huber in 1972 [227], further characterized by Devlin in 1977 [30], and synthesized from l-cocaine in the same year providing a pure and abundant source of the toxin for toxicological studies [228].

Anatoxin-a binds to nicotinic acetylcholine receptors with the same affinity or greater as acetylcholine. Structurally, anatoxin-a is a bicyclic amine alkaloid, similar to that of epibatidine, but lacking the peperidine motif [229]. Upon binding irreversibly to acetylcholine receptors, anatoxin-a causes depolarization of postsynaptic neuronal cells, or efflux of Ca^+^ and Na^+^ ions, generating an action potential. Acetylcholinesterase does not degrade the anatoxin-a-acetylcholine receptor complex. Thus, the depolarized state becomes permanent and the nerve is desensitized. Anatoxin-a also apparently affects the presynaptic nerve reducing the frequency and quantal content of miniature end plate potentials [230]. Symptoms begin with convulsions eventually leading to paralysis and death due to suffocation by respiratory arrest. The positive optical isomer (+)-anatoxin-a acts as an agonist of the acetylcholine receptor at concentrations orders of magnitude below that of (−)-anatoxin-a and is five times more lethal than a racemic mixture of (+/−)-anatoxin-a [231,232].

The reported dose of anatoxin-a causing lethality via IP injection in mice varies widely. Fawell et al. [233] report that 100% of animals died receiving anatoxin-a at 100 µg/kg·b.w. (within 1 min) and at 60 µg/kg·b.w. during Erwin and rotarod tests, respectively. Carmichael et al. [225] reported 300 µg/kg·b.w. as the minimum lethal dose. Rogers et al. reported that 300 µg/kg·b.w. caused all animals to die, and 250 µg/kg·b.w. was the 50% lethal dose. In any case, the LD_50_ via oral gavage is orders of magnitude higher at >10,000 µg/kg·b.w. [233,234,235]. Recovery from exposure to sub-lethal concentrations is reported to be quick with no lasting, long-term effects in mice given sub-lethal concentrations [233,236]. A methylated variant of anatoxin-a, homoanatoxin-a was synthesized as a homolog for structure-activity studies [237], then subsequently found to be produced naturally in an *Oscillatoria* species [238]. Homoanatoxin-a has an identical mode of action and similar potency as anatoxin-a [238]. The 4-hydroxyanatoxin-a analog is an oxygenated variant of homoanatoxin-a produced by *Raphidiopsis* (or probably *Cylindrospermopsis* [239]) and is apparently non-toxic [240,241].

Carmichael et al. [225] observed that different strains of *Ana. flos-aquae* produce different symptoms in the mouse. Some strains caused toxicity similar to that of anatoxin-a, but with the added symptom of salivation. Thus, Mahmood et al. [242,243] described another neurotoxin from *Ana. flos-aquae* NRC 525-17, previously isolated from Buffalo Pound Lake Saskatchewan, Canada in 1965. The toxin was named anatoxin-a(S) where the “s” indicates salivation. Upon injecting mice with an extract of *Ana. flos-aquae,* mice died of respiratory arrest within minutes following convulsions. However, the toxin did not mimic acetylcholine. Instead, anatoxin-a(S) was found to cause acetylcholine accumulation by inhibiting acetylcholinesterase in both muscarinic and nicotinic junctions. Thus, infusion with anatoxin-a(S) results in marked declines in heart rate and blood pressure, prior to decreases in respiratory volume [244]. The inhibition of acetylcholinesterase by anatoxin-a(S) is irreversible. Mice that do not die given 290 µg/kg·b.w. anatoxin-a(S) by IP injection show inhibition of red-blood cell cholinesterase for at least 8 days accompanied by twitching and fasciculations [36]. Thus, symptoms at sub-lethal concentrations may be prolonged.

The structure of anatoxin-a(S) is unrelated to anatoxin-a and was found to be a unique phosphate ester of a cyclic *N*-hydroxyguanidine [243]. The structure was in agreement with previous studies showing that similar structures, namely esters of *N*-hydroxysuccinimide, are effective in inactivating acetylcholinesterase [245]. The LD_50_ of anatoxin-a(S) via IP injection in mice is considerably lower than that of anatoxin-a at 20 µg/kg·b.w.; while in rats, a 9 µg/kg·b.w. dose has been shown to be consistently lethal within 1 h [37,243]. Overt clinical signs including behavioral deficits are observed at 1.5 µg/kg·b.w. [36,242,243,246,247]. By comparison, another acetylcholinesterase inhibitor, paraoxone insecticide, caused clinical symptoms at 800 µg/kg·b.w. in rats [37]. Anantoxin-a(s) is a direct agonist at muscarinic sites with indirect neuromuscular blockade [244]. Thus, the effects of anatoxin-a(S) can be blocked at least temporarily with atropine [244].

For the most part, the anatoxins are associated with only acute illness with no connections to long-term neurotoxic illness. However, some recent studies have suggested possible developmental effects of anatoxin-a or effects of anatoxin-a at lower concentrations in repeat doses in mammalian cells, amphibians, and fish. Rogers et al. show perturbations in mouse yolk sac in an in vitro assay at ~165 µg/L and above and dose-dependent transient narcosis, edema, and loss of equilibrium in toad embryos. Rymuszka et al. [248] show immune cell cytotoxicity in carp exposed to 25 µg/L by immersion. Despite these studies, there appears to be little evidence for chronic effects of anatoxin-a at sub-lethal/low-dose concentrations or developmental effects in utero. However, there are few studies that have examined more subtle pathological changes (e.g., epigenetics). In addition, there are few or no studies that have examined the contribution of anatoxin-a(S) to chronic disease, and it should be noted that commercial or academic sources of anatoxin-a(S) disappeared after the 1990’s, at least within the U.S. Thus modern toxicological studies of anatoxin-a(S) are currently non-existent. 

At least three studies on the oral toxicity of anatoxin-a have been conducted. Stevens and Krieger [234] determined that the oral LD_50_ of synthetic anatoxin-a in mice using a single oral dose is 16,200 µg/kg·b.w. However, the method of delivery is not given and no other endpoints were measured. In a study by Astrachan and Archer [236,249] rats were exposed to a partially purified extract of anatoxin-a producing *A. flos-aquae* for seven weeks at dosages of 500 and 5000 µg/kg·b.w. No changes in body weight, food consumption, serum liver enzymes and gross pathology were observed. Increased white blood cell counts were observed for 5 weeks. Based on this the authors indicated a NOAEL of 50 µg/kg·b.w./day. However, since a partially purified extract was used it is not clear if the effects are due to anatoxin-a or other cyanobacterial metabolites present in the crude extract. 

Fawell et al. [233] exposed male and female mice to anatoxin-a for 28 days via oral gavage. Ten male and ten female mice in each exposure group were given doses of 0, 98, 490, and 2460 µg/kg·b.w./day anatoxin-a. Mice were examined daily for clinical signs of illness, body weights were measured weekly and in the final week of the study blood hematology and serum chemistry were characterized. In addition, tissues from the control and high dose group were examined microscopically. There were no dose-related, statistically significant changes in any of the parameters measured. Three animals died during the study, but without any symptoms and one death occurred due to fighting among mice in the same cage. A NOAEL of 98 µg/kg·b.w. per day is suggested, but it is not clear what endpoint that NOAEL is based upon.

### 4.3. Cylindrospermopsin

Cylindrospermopsin has been implicated in several human poisoning events [250]. In 1979, an outbreak of severe gastrointestinal illness with symptoms of acute liver failure occurred on Palm Island, Northern Queensland, Australia [251,252]. The outbreak was associated with the local water supply at Solomon Dam, which had been treated with copper sulfate known to lyse cyanobacterial cells releasing toxins and other cellular constituents. *Anabaena* and *Cylindrospermopsis* were the two major genera present in source waters at the time illnesses occurred [253]. Strains of these two genera were isolated and tested for toxicity [254]. The *Anabaena* strain proved to be non-toxic while IP injection of mice with lyophilized culture of the *Cylindrospermopsis* strain caused death within hours (LD_50_ = 64 mg/kg) [254]. The liver was primarily affected with massive hepatocyte necrosis. In addition, lesions were produced in kidneys, adrenal glands, lungs, and intestines. Prior to this event, *Cylindrospermopsis* species were presumed to be non-toxic.

Ohtani et al. [255] purified a toxin from *Cylindrospermopsis* and characterized its structure in 1992. Subsequently, cylindrospermopsin was also detected in cultures of the cyanobacterium *Umezakia natans* (Nostocaceae) [256]. Norris et al. suggested that cylindrospermopsin alone does not account for the toxicity of *Cylindrospermopsis* extracts. In attempts to resolve this, they identified and characterized an analog of cylindrospermopsin in *Cylindrospermosis* extracts, deoxycylindrospermopsin. This compound is produced in various quantities by *Cylindrospermopsis* alongside cylindrospermopsin. It appears this analog is non-toxic in mice via IP up to 800 µg/kg·b.w. On the other hand, recently, Neumann et al. [257] show that deoxycylindrospermopsin inhibits protein synthesis at the same potency as cylindrospermopsin in an in vitro model. In addition, 7-epicylindrospermopsin is another analog of cylindrospermopsin that is toxic, but has so far only been found in *Aphanizomenon* and *Oscillatoria* species as minor metabolites [258]. Breakdown products and analogs of cylindrospermopsin have been found in drinking water systems including cylindrospermic acid and chlorocylindrospermopsin. Based on a study by Looper et al. [259] it appears that the oxygenation at the C7 position of this toxin is not required for toxicity (i.e., protein synthesis inhibition). Thus, the native molecule and all analogs detected so far including breakdown products detected during drinking water purification are toxic.

The LD_50_ in mice of cylindrospermopsin over a 24-h time period via IP is 2000 µg/kg·b.w., but over 5 days the LD_50_ is 200 µg/kg·b.w. (similar to anatoxin-a) [255]. Cylindrospermosin toxicity is still under investigation, but likely occurs by multiple mechanisms. In hepatocytes, cylindrospermopsin has been shown to cause a decrease in glutathione levels, inhibit of protein synthesis, membrane proliferation, fat droplet accumulation, and decreased levels of P450 enzymes [260,261]. Overall, the toxicity of cylindrospermopsin follows three mechanisms, which may be interacting: (1) cytochrome P450-metabolism dependent reactive oxygen species (ROS) production, (2) inhibition of protein synthesis, and (3) genotoxicity. 

In the presence of cytochrome P450 inhibitors (piperonyl butoxide), mice are completely protected from death given cylindrospermopsin up to 800 µg/kg·b.w. [262]. Thus, P450 plays a critical role in the toxicity of cylindrospermopsin. Cytochrome P450 enzymes are oxygenases responsible for metabolism of xenobiotic compounds. The activities of these enzymes with certain substrates have been shown to contribute to the production of ROS, mutagenicity, and cellular toxicity, particularly in liver disease. Treatment of human hepatoma cells with less than 10 µg/L cylindrospermopsin induces ROS production. ROS production may also be induced by metabolites of cylindrospermopsin. In mice administered 200 µg/kg·b.w. cylindrospermopsin, 23% of cylindrospermopsin is retained in the liver for up to 2 days. In addition, both methanol extractable and non-methanol extractable metabolites of cylindrospermopsin are retained in the liver. ROS production accompanied with a reduction in reduced glutathione is problematic since glutathione has a central role in protection against ROS [263]. However, Humpage et al. [264] report that malondialdehyde, a marker of oxidative stress, did not increase in the presence of cylindrospermopsin, suggesting that ROS may not be the mediator of cylindrospermopsin cytotoxicity. They also show that cylindrospermopsin, rather than phase 1 products of cylindrospermopsin metabolism by P450, is the primary acutely acting cytotoxin and not cylindrospermopsin breakdown products. 

Cylindrospermopsin has been shown to inhibit protein synthesis at the elongation step in both plant and mammalian cell extracts [265]. Furthermore, the toxin has been shown to bind non-covalently to components of the translational machinery that does not include the ribosomes [256,265]. This suggests cylindrospermopsin binds to Eukaryotic initiation factors. While the exact mechanism of protein synthesis inhibition is not completely understood it appears to be the primary mode of toxicity at lower doses of cylindrospermopsin, while at high doses cytochrome P450 metabolism of cylindrospermopsin producing ROS induces cell death [26,261].

Cylindrospermopsin is associated with genotoxicity. Falconer and Humpage [266] show that mice treated with cylindrospermopsin by oral gavage showed 5 tumors out of 53 treated animals compared to none in the control group. Treatment of immortalized human cell lines with 1–10 µg/L cylindrospermopsin has been shown to cause the production of micronuclei and DNA damage [267]. Furthermore, comet assays to detect DNA breaks were positive for cylindrospermopsin treatment in a lymphoblastoid cell line, where inhibitors of P450 (omeprazole and SKF525) were protective against genotoxicity. Straser et al. [268] show that cylindrospermopsin caused the up-regulation of genes affecting the P53 tumor suppressor protein. P53 is involved in cell cycle arrest particularly during the course of DNA damage. MDM2 and CDKN1A have been shown to negatively regulate P53 [269,270]. In cylindrospermopsin treated cells, expression of both of these proteins is increased [269] suggesting lack of cell cycle arrest during DNA damage by cylindrospermopsin. 

Based on 10 and 11-week oral exposure studies in mice, a NOAEL of 30 µg/kg·b.w. per day was indicated by Humpage and Falconer [264,271] suggesting a maximum acceptable concentration for drinking water of 1 µg/L. In this study, two experiments were carried out, but only the second experiment used purified toxin. Male Swiss albino mice were given purified cylindrospermopsin by oral gavage daily at 0–240 µg/kg·b.w. The cylindrospermopsin was purified from cell lysate by solid phase extraction on a C18 cartridge followed by size exclusion using SephadexG10, and preparative HPLC purification on a C18 column. The purified product was characterized by time-of-flight mass spectrometry and nuclear magnetic resonance spectroscopy. These analyses showed that the final yield of cylindrospermopsin was 10.4 mg in a total of 22 mg, and that at least one of the major contaminants was phenylalanine. Thus, the purity of cylindrospermopsin used was 47%. The solvent used to dissolve the purified cylindrospermopsin before dosing (e.g., methanol or water) was not reported and could be a confounding variable. 

Clinical examinations of the mice were performed throughout the exposure period. This included examination of skin, fur, eyes, mucous membranes, respiration, pupil size, gait, posture, grooming behavior pattern, noise response, visual response, touch response, grip strength, motor activity, and evidence of lachrymation or abnormal excretions. Following the trial, serum and urine chemistry were characterized, hematology was performed, body and organ weights were determined, and a complete histological examination of all organs was performed.

**Figure 12:**
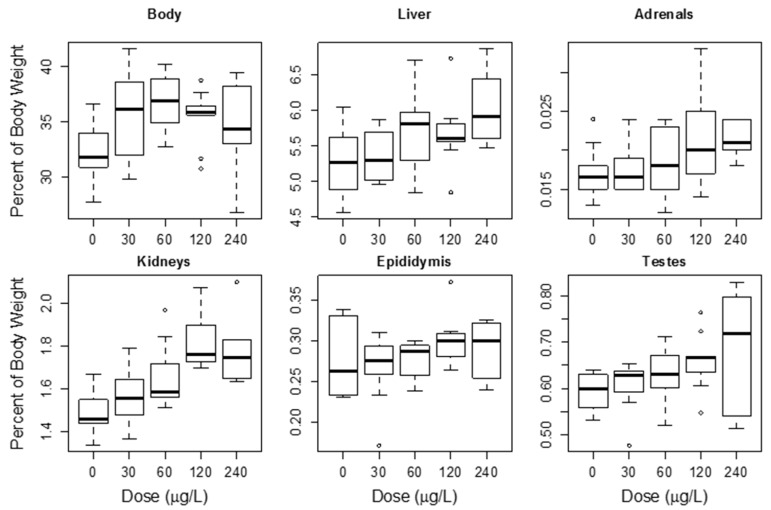
Increase in organ and body weights due to oral exposure to 0–240 µg/kg·b.w. cylindrospermopsin. Data from Humpage and Falconer 2002.

No clinical changes in appearance or behavior were observed at any exposure level, except for a reduction in water consumption at all levels at 53–72% of the control in the last four weeks of the experiment. There was also no visible organ or tissue damage upon post-mortem examination and no significant changes in serum liver enzymes. The most significant changes were seen in body and organ weights expressed as a percentage of body weight. There was a significant increase in body weights in the 30 and 60 µg/kg·b.w. doses (Figure 12), but this trend did not continue in the higher doses. Organs that followed a positive increasing trend with dose were liver, adrenal glands, kidneys, epididymis, and testes. Kidney weight was significantly increased at all doses at and above 60 µg/kg·b.w. Liver weight was significantly increased in the 240 µg/kg·b.w. dose and if not for two outliers was also significantly increased at the 120 µg/kg·b.w. dose. A dose-dependent increase in mitotic figures, inflammatory foci and necrotic cells was observed in liver sections, but not in other organs.

Since kidney weight was increased in a dose-dependent manner at 60 µg/kg·b.w. and higher the NOAEL for cylindrospermopsin kidney toxicity was determined to be the next lowest dose at 30 µg/kg·b.w. where no adverse dose dependent effects were observed given the endpoints measured. Humpage and Falconer calculated a drinking water guideline of 1 µg/L. To do this a tolerable daily intake level of 0.03 µg/kg·b.w./day was estimated by dividing the NOAEL by an uncertainty factor of 1000. The uncertainty factor included a multiplier of 10 for lack of sufficient data on cylindrospermopsin toxicity, and 100 for inter-and intraspecies variability for a product of 1000. A drinking water guideline value was calculated by multiplying the tolerable daily intake level of 0.03 µg/kg/day by the weight of an adult (60 kg) and the proportion of drinking water from tap water (0.9) divided by the assumed tap water intake for an adult (2 L/day).

### 4.4. Saxitoxin

Voltage-gated ion channels are found in neuronal and muscle tissue and are responsible for the rising phase and propagation of action potentials in excitatory cells [272]. A variety of organisms in nature produce molecules that block, activate, or modulate these ion channels [273]. Among these, saxitoxins and structurally similar compounds (gonyautoxins) bind to voltage gated sodium channels preventing the flow of sodium ions and action potentials [274,275,276]. Saxitoxin also modifies the voltage gating processes in K^+^ channels and blocks l-type Ca^2+^ channels [277,278]. Thus early symptoms of saxitoxin poisoning are tingling or burning of the lips, tongue and throat. Later symptoms include complete numbness of the face followed by paralysis. Death usually occurs due to suffocation or cardiac arrest.

Saxitoxins and its analogs are collectively known as paralytic shellfish poisons (PSPs) and are widely distributed in nature occurring in bacteria, cyanobacteria, marine algae (dinoflagellates), shellfish, fish, worms, Echinoderms, and crustaceans among others (reviewed in [279]). Some of the earliest investigations of PSPs were focused on identifying the source of toxicity in shellfish, primarily in mussels due to outbreaks of PSP poisoning in California affecting hundreds of people [280]. In 1937 Sommer et al. [281] presented evidence that the source of toxicity in mussels was associated with algal cells and that toxicity was highest when large numbers of certain algae, dinoflagellates, were present in the water column. Furthermore they purified paralytic toxins from field collections of *Gonyaulax catenella*. Genes for saxitoxin synthesis have now been discovered in the dinoflagellate *Alexandrium* and several genera of cyanobacteria [282,283]. This definitively proves that these organisms synthesize saxitoxin de novo. In other organisms it is thought that saxitoxin and other PSPs accumulate within animal tissues, either due to the presence of dinoflagellate/cyanoacterial symbionts, or due to the consumption of these PSP producers, rather than synthesizing the toxin themselves [284]. In fact, some PSP containing animals contain saxitoxin binding proteins or transferrins known as saxiphillin [279]. The saxiphillin allows saxitoxin to accumulate to high levels within animal tissues without causing toxicity since the saxiphillin competes with saxitoxin binding to Na^+^ channels [285]. 

This is a potential human health hazard. For example, puffer fish contain saxiphillins allowing them to accumulate high levels of saxitoxin in certain organs, primarily the liver [286]. Consumption of puffer fish has led to human illness and fatalities [287]. Saxiphillins are widely distributed in nature occurring in ecologically diverse species including fish, reptiles, and amphibians including the American bullfrog [285].

Saxitoxin potency in mice is higher than that of most other cyanotoxins discussed above. Using a purified preparation from shellfish, Wiberg and Stevenson [288] show that the oral, IP, and intravenous LD_50_ for saxitoxin in mice is 236 µg/kg, 10 µg/kg, and 3.4 µg/kg, respectively. Furthermore, the dose response curve was steep, as the difference between the dose killing 50% vs. 99% of animals was only 6 µg/kg. Using purified toxin, Kao and Nishiyama [276] found that IP injection of 2 µg/kg resulted in severe neuromuscular paralysis and hypotension while only 0.75 µg/kg produced paralysis without hypotension. In addition, the authors noted that placing droplets of toxin at 0.01 µg/mL (10 µg/L) on their lips and hands resulted in paraesthesia and numbness. Based on reports of known human illness due to saxitoxin or PSP exposure in ~500 individuals the European Food Safety Authority, Panel on Contaminants in the Food Chain (CONTAM) estimated a NOAEL for saxitoxin of 0.5 µg saxitoxin equivalents per kg·b.w., translating to a 30 µg dose for a 60 kg individual [289]. 

Synthesis and transformation of saxitoxins and PSPs leads to structural diversity and therefore a range of toxicities. PSPs are broadly defined as hydrophobic or hydrophilic, and may contain side chains including sulfate, acetate, carbamoyl, hydroxyl, or hydroxybenzoate groups. Those PSPs with carbamoyl side chains appear to have the greatest affinity for their ligands whether ion channels or saxiphillins [285]. Once released from dinoflagellates or cyanobacteria the PSPs may be transformed by associated heterotrophic bacteria. For example, bacteria isolated from mollusk tissue have been shown to transform gonyautoxins types 1 and 4 to types 2 and 3. Furthermore, other isolates were able to decarbamoylate or sulfate gonyautoxins [290]. In addition, Kotaki et al. [291] show that *Vibrio* and *Pseudoalteromonas* species isolated from crabs and a grastropod converted gonyautoxins to saxitoxin and neosaxitoxin.

In the Great Lakes region *L. wollei* has been shown to produce decarbamoylsaxitoxin and decarbamoylgonyautoxin-2 and-3 as well as six novel saxitoxins [54,55]. According to mouse bioassays these appear to be at least 10 fold less toxic than saxitoxin [285]. However, as mentioned previously there is the possibility that *L. wollei* saxitoxins could be converted to other more toxic PSPs by bacteria. In addition, the potential saxitoxin producer *C. raciborskii* has been detected in the Great Lakes region [29]. While no *Aphanizomenon* species from the Great Lakes region have been shown to produce saxitoxins (or aphantoxins), blooms of *Aphanizomenon* species in other north temperate regions of the world have been shown to produce saxitoxins [292] and *Aphanizomenon* is a common bloom forming species in the Great Lakes region [126,293]. Thus there is considerable concern that with increased eutrophication saxitoxins could become a greater human health concern in the Great Lakes region.

## 5. Numerical Limits

In order to protect public health it is necessary to establish the maximum cyanotoxin concentration and duration of exposure, below which no adverse health outcome is expected. In 2007 and again in 2012 Chorus [294] reviewed cyanotoxin risk management plans and regulations developed by various countries. At least six countries including Canada have developed regulations establishing maximum acceptable concentrations, or provisional guideline values for select cyanotoxins and/or cyanobacterial biomass in either recreational and/or finished drinking water systems. In the United States there are no federal regulations limiting cyanotoxin concentrations in finished drinking water, however, some individual states, including Oklahoma have developed their own regulations for recreational waters. 

A lack of regulation is due in part to uncertainty over what cyanotoxin concentration and duration of exposure is considered safe. As a first step, the United States Environmental Protection Agency (USEPA) placed cyanobacteria biomass (draft 1) or cyanotoxins (anatoxin-a, MCLR, and cylindrospermopsin) on their Contaminant Candidate Lists since 1998 (draft 1 and 2) and 2009 (draft 3), respectively. Draft 4 to be released soon is limited to these three cyanotoxins (e.g., saxitoxin is not listed). In some cases, it may be possible to derive numerical limits based on the most current literature. Below we discuss numerical limits for the most common cyanotoxins in the Great Lakes region for which there is sufficient toxicological data.

### 5.1. Microcystin

The majority of countries with some form of regulation or established health advisories follow closely to the WHO provisional guideline value of 1 µg/L/day MC in drinking water. This limit is based on the tolerable daily intake level of 40 µg/kg·b.w. per day determined by Fawell et al. [155] and Falconer et al. [156], a mean body weight of 60 kg, and a 1000 fold uncertainty factor. The uncertainty factor includes a factor of 100 for intra-and interspecies variation in toxicity as well as a factor of 10 for lack of information on MC toxicity, particularly chronic exposures. It was also assumed adults drink 2 L of water per day and that 80% of this is from tap water. Thus, this guideline is primarily directed at adults. 

Children as opposed to adults have most often been the victims in human poisoning events [295,296] and are more at risk for illness as a result of cyanotoxin exposure given a relatively smaller body size proportional to the volume of water they consume and the potential for developmental toxicity [297,298,299,300]. Weirich and Miller [297] recalculated the WHO guideline value adjusting the mean body weight to that of children aged 9 months, 5.5, or 9.5 years. In addition, daily drinking water intake was adjusted to 1 L for all ages. Based on this calculation for children aged 5.5 years and 9 months the guideline value was reduced to 0.6 and 0.3 µg/L, respectively. 

The USEPA recently published a health advisory for MCs in drinking water [301]. They established provisional guideline values for two different age groups, (1) 0.3 µg/L for bottle-fed infants and pre-school aged children under 6 years of age, and (2) 1.6 µg/L for children older than 6 years of age and adults. Both are ten-day guideline values, meaning that exposure for ten days below these levels is considered protective of public health, but effects of exposure at any level for longer than 10 days is unknown. The guideline values were calculated by dividing a lowest-observed-adverse effect level (LOAEL) of 50 µg/kg·b.w./day by the product of an uncertainty factor (1000) multiplied by the drinking water intake rate (L/day) normalized to mean body weight of each age group. 

Reference dosages such as the LOAEL/NOAEL are typically taken from animal exposure studies. While a number of animal studies have described the toxicity of MCs, relatively few studies have quantified toxicity of MC in a rodent model using pure compound and in well-described, controlled repeat oral dose trials at multiple exposure levels. Oral exposure compared to other exposure routes (e.g., IP injection) in animal models, mimics human exposure to cyanotoxins in drinking water, as well as ingestion recreationally. In addition, use of pure compound as opposed to mixtures or crude cell extracts is necessary in order to establish health effects of each compound independently, though research is needed on the effects of defined cyanotoxin mixtures. 

A NOAEL of 40 µg/kg·b.w./day derived from Fawell et al. [155] and supported by a separate study by Falconer et al. [156] was used by the WHO in calculating their provisional guideline level. The LOAEL for calculating the EPA guideline value for MCs was based on the study by R. Heinze in 1999 [157]. The USEPA advisory for MCs used this study to determine that the LOAEL for MCLR is 50 µg/kg·b.w./day via the oral route with the major end point being liver lesions. The study by Heinze did not test lower dosages to determine if they would also cause liver damage, so based on that study alone it is not known if 50 µg/kg·b.w./day is the lowest dose causing liver lesions. Modeling the dose-response relationship might provide evidence that lower doses would produce no or minimal adverse effects, but with only two exposure groups it would be difficult to establish a statistically significant dose-response curve. As such, the USEPA applied an uncertainty factor of 3 to account for extrapolating from a LOAEL to a NOAEL, meaning that the NOAEL could be as low as 16.7 µg/kg·b.w./day. This may not be sufficient for other adverse effects reported for MCs including reproductive and neurotoxic effects as described above.

A LOAEL of 50 µg/kg·b.w./day is at least partially corroborated by the Fawell et al. study, which found no significant pathological changes in the liver at 40 µg/kg·b.w./day MCLR. Yet, the Fawell and Heinze studies are not directly comparable because the Fawell et al. study used mice, not rats, in their 13-week repeat oral dose study. In addition, mice were given MCLR by oral gavage whereas the Heinze study provided five rats per cage with 150 mL/day of drinking water containing MCLR, 93–97% of which was consumed by the animals. The Heinze study was also for a shorter duration (28 days), which might be more realistic for calculating a 10-day health advisory.

**Figure 13:**
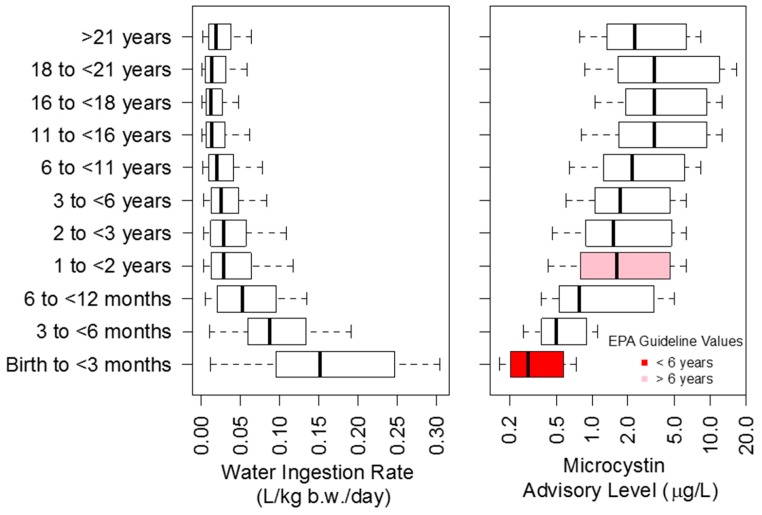
Variability in drinking water ingestion rates across all age groups and percentiles (**left**) and resulting microcystin advisory levels for children under six calculated using drinking water ingestion rates (**right**). Note, the right x-axis is on a log scale. The middle black bar in each box represents the median, and the box width represents the upper and lower quartiles.

The EPA guideline value takes into account the drinking water intake rate by mean body weight ratio (DWI/BW) of each age group. These values were derived from existing data. For children <3 months old the data used by EPA were from the USDA Continuing Survey of Food Intakes by Individuals program 1994–1996, and 1998 [302], whereas data for all other age groups were from the National Health and Nutrition Examination Survey 2003–2006 [303]. Young children as opposed to teens and adults have a higher daily water intake rate per kg of body weight. For example, infants <3 months old have a DWI/BW over ten times that of 16 to 18 year olds (Figure 13). Unless there is some reason to expect that infants and children will not be consuming municipal drinking water, the guideline values for cyanotoxin exposure need to be directed at the most sensitive age groups.

The USEPA advisory uses DWI/BW data at the 90th percentile of usage to calculate the guideline values. The resulting values for various age groups then ranges from 0.2 µg/L for infants to 2.2 µg/L for 3–6 year olds at this 90th percentile level. A guideline value of 0.3 µg/L was chosen instead of 0.2 µg/L for children <6 years of age. The EPA advisory states that the uncertainty factor of 10 for intraspecies variation adequately accounted for this difference and reflects the average DWI/BW ratio of children under 6. That uncertainty factor included 10 for inter-and 10 for intraspecies variability, 3 for lack of data, and 3 for extrapolating from a LOAEL to NOAEL.

The percentile category of DWI/BW data used affects the calculated guideline value considerably. For example, for children under 3 months of age, arguably the most sensitive population, the calculated guideline value ranges from 0.16 to 4.34 µg/L depending on if DWI/BW data are taken from the 99th or 10th percentile respectively (Figure 13). Thus, using the EPA formula, the most conservative guideline value would be 0.16 µg/L for bottle-fed infants.

There is some question as to whether a provisional guideline value can be applied to children and infants when the LOAEL used to calculate that guideline value was derived from a study of adult male rats. There is some evidence in the literature for potential developmental effects of MCs [304,305]. As such it is possible that individuals of a younger age could develop adverse effects later in life due to an exposure at levels lower than 50 µg/kg·b.w./day, during critical periods of development.

Liver damage may not be the only adverse health effect end point to consider when developing numerical limits for MCs. While the liver is clearly one of the most affected organs, MCs also affect brain and reproductive tissues [158,159,160,161,162,163,164,165,166,167]. 

The study by Li et al. reported central nervous system toxicity after repeat oral dose exposures of MCLR in rats. The EPA health advisory does not consider this study in its formulation of the drinking water guideline value for MCs or neurotoxicity of MCLR as an end point. One problem with the Li et al. study is that the MCLR stock (1 mg) was dissolved in 1 mL methanol to a concentration of 1000 µg/mL, then diluted ten-fold with pure water to a working stock concentration of 100 µg/mL. At that point the working stock was 10% methanol. This working stock was then diluted with water to produce drinking water at 0.2, 1.0, and 5.0 µg/mL or 0.02%, 0.1%, and 0.5% methanol. Thus in addition to varying MCLR dosage, rats received varying levels of methanol, a known neurotoxin [306,307]. By comparison, the Heinze study dissolved 20 mg MCLR in absolute ethanol (10,000 µg/mL) to make a stock solution, this was diluted in pure water to 1000 µg/mL working stock (10% ethanol) and diluted further by an unknown amount to produce 150 mL of MCLR laden drinking water for 5 rats. The use of ethanol over methanol as a solvent is beneficial as ethanol is much less toxic than methanol [308]. 

Li et al. [170] also examined the developmental effects of MCLR in a repeat maternal oral dose study in rats showing deficiencies in the Morris water maze test of offspring due to MCLR exposure in utero. As with Li et al. (2014), the study by Li et al. (2015) used methanol to make the stock solution of MCLR resulting in trace levels of methanol in all exposures. However, in the Li et al. (2015) study methanol was included in the negative control and was normalized to 0.002% (i.e., 1.6 mg/kg·b.w.) in all treatments including the control. The EPA health advisory for MCs does not consider this study in calculating the guideline value for MCs in drinking water. The EPA health effects support document [298] indicates that the study is confounded because there may be synergy between methanol exposure and MCLR.

Reproductive toxicity is another endpoint to be considered in formulating guideline values. Chen et al. [171] show effects of MCLR on male reproductive tissues (e.g., sperm motility/counts, testis weight) in repeat dose oral exposure studies in mice. The EPA health advisory considered this study in deciding whether to include reproductive effects as an endpoint, but determined that the study design was lacking in several ways. The EPA indicates that no testis weights were given, no methods were given for how sperm was handled, or how percent sperm motility was determined, the purity of MCLR, species and ages of mice, body weight, amount of water consumed, and dosage levels. 

Chen et al. indicate that sperm counts and motility were determined using the HTM-TOX IVOS semen analyzer and that MCLR was purchased from Alexis Biochemicals (Enzo Life Sciences), which sells MCLR at >95% purity. The species of mouse is not given, but their body weights are given (15–25 g) which would allow one to estimate the exposure level. Water intake across 28 mouse strains ranges from 4 to 8 mL per day [309]. The lowest level at which effects were observed was at the 3.2 µg/L level. Therefore, at this exposure level the dosages were likely between 0.9 and 1.7 µg/kg·b.w./day for a 15 g mouse consuming between 4 and 8 mL per day, or between 0.5 and 1.0 µg/kg·b.w./day for a 25 g mouse consuming between 4 and 8 mL per day, an overall mean estimated dosage of 1.0 µg/kg·b.w./day at the 3.2 µg/L level. The EPA Health Support Document for MCs calculates a LOAEL and NOAEL of 0.79 and 0.25 µg/kg·b.w./day for reproductive toxicity based on this study. This suggests that adverse effects caused by MCLR on reproductive tissues occurs at doses well below the LOAEL for liver toxicity of 50 µg/kg·b.w./day.

If the guideline values were calculated for endpoints of reproductive and neurotoxicity using the aforementioned studies then they would be lower than guideline values based on liver toxicity. Using the Chen et al. [171] study for a reproductive toxicity LOAEL (1.0 µg/kg·b.w./day), and the Li et al. study for a neurotoxicity LOAEL (5 µg/kg·b.w./day) the guideline value at the 90th percentile DWI/BW ratio ranges from 0.004 to 0.029 µg/L/day for reproductive toxicity and 0.02–0.15 µg/L/day for neurotoxicity across age groups from <3 months to adults. The mean guideline values for children <6 years old would be 0.01 µg/L and 0.07 µg/L for reproductive and neurotoxicity, respectively (Table 4). These are 4- and 30-fold lower than the EPA guideline value for liver toxicity in children <6 years old. Given these large differences it is critical that future studies confirm the level of MC that displays reproductive and neurotoxicity in repeat oral dose study designs. 

EPA and WHO guideline values are all based on studies of the toxicity of the MCLR congener. However, MC congers vary widely in their potency from those that are non-toxic to those that are as toxic or more than MCLR based on in vitro assays or animal studies [310,311,312,313]. As discussed above the range of potency displayed by MC congeners is primarily due to (1) recognition and transport rates of different MC congers by OATP receptors and (2) presence/absence of an intact MDHA moiety at position 7 in different MC congeners affecting binding to cysteine residues in phosphatases [314,315]. Thus, guideline values based only on studies of MCLR toxicity may not reflect the true toxicity of mixtures of MC variants that occur in nature. 

Monitoring and analytical methods for MC detection will necessarily be influenced by the drinking water guideline value. The EPA advisory suggests MCLR is a suitable surrogate for the toxicity of all MCs because it is one of the most commonly occurring or monitored in the environment and is one of the most toxic congeners. Furthermore, the EPA advisory indicates that the guideline values apply to total MCs in a given sample. MCs can exist bound to proteins or other molecules with thiol groups (e.g., free cysteine), transformed, or otherwise in a non-toxic state. The guideline value should be applied to the total toxic MCs (TTMC), which would include non-transformed, non-protein bound total MCs capable of covalently binding to and inhibiting protein phosphatases. Methods for specifically quantitating the TTMC pool in drinking water have not been demonstrated. In theory, the MC ELISA would measure non-toxic protein bound as well as toxic, non-protein bound MCs. The EPA advisory suggests using EPA Method 544 for quantification of intracellular and extracellular MCs using LC-MS/MS in drinking water. However, this method does not target TTMCs and the surrogate standard indicated for use in that method is no longer available. The PP1/2A inhibition assay might be useful as an activity assay in quantifying TTMCs, but it would also detect other PP1/2A inhibitors mentioned above. Further complicating this issue is the fact that a recent study shows MCs covalently bound to thiols can deconjugate over time releasing a fully intact MC molecule. If the guideline value is for TTMCs then method development for detecting these compounds is critical. 

### 5.2. Cylindrospermopsin

Some countries have developed regulations and/or advisories for cylindrospermopsin in drinking water including Brazil [316]. The USEPA recently developed cylindrospermopsin guideline values for drinking water [317]. The 10-day guideline values are directed at two age groups, (1) 0.7 µg/L for pre-schooled aged children <6 years old and (2) 3 µg/L for children over 6 years of age and adults. As with MCs, unless it can be guaranteed that children under six years old will not be consuming the water then drinking water plant operators would need to follow the lower value.

**Figure 14:**
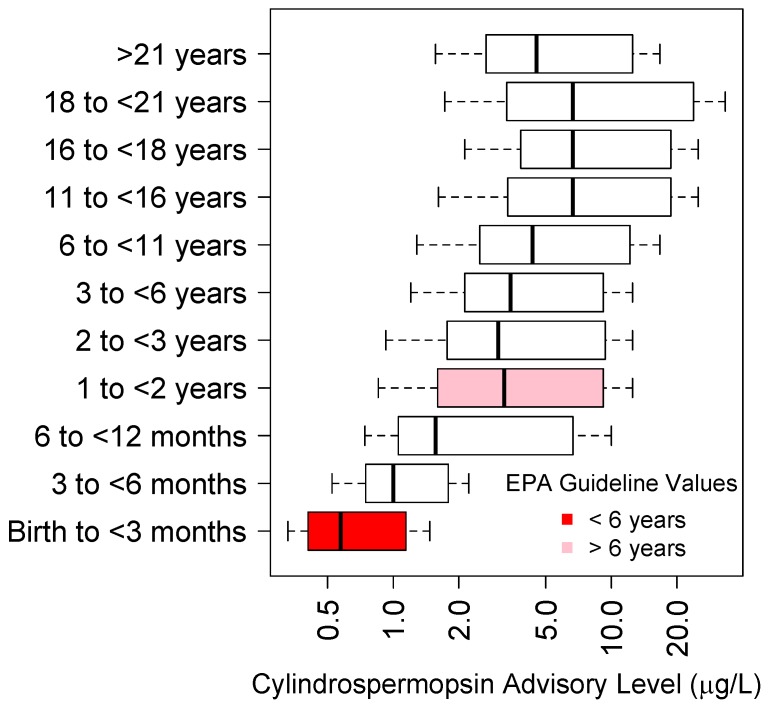
Variability in calculated cylindrospermopsin advisory levels at all age levels and drinking water ingestion rates using a NOAEL of 30 µg/kg.

The EPA guideline value for cylindrospermopsin was calculated using the same formula as for MCs and a NOAEL of 30 µg/L/day from Humpage and Falconer [264]. The calculation of the EPA cylindrospermopsin guideline value differs from the Humpage and Falconer calculation in the use of a lower uncertainty factor and use of drinking water intake rate normalized to body weight of a specific age group (over the first year of life). The uncertainty multiplier used for inter-and intraspecies variation was the same as Humpage and Falconer at 100, but the uncertainty multiplier for lack of data on cylindrospermopsin toxicity was lowered from 10 to 3. This resulted in an overall uncertainty factor for the cylindrospermopsin guideline value of 300. It could be argued that the more conventional multiplier of 10 for lack of data should be applied. There have been no other oral exposure studies of cylindrospermopsin toxicity similar to the Humpage and Falconer study since 2003 and as stated in the EPA support document for cylindrospermopsin [299] “no oral reproductive or developmental and chronic toxicity studies are available for cylindrospermopsin.” In addition, the advisory states that there is a lack of data on any potential neurological effects. Finally, an uncertainty factor of 1000 is warranted for cylindrospermopsin because the guideline value is essentially based on one experiment and no replicate experiments. With an uncertainty factor of 1000 the guideline value changes to 0.1–0.9 µg/L for age groups from <3 months to adults over 21 years old, respectively at the 90th percentile of DWI/BW ratio (Figure 14), or 0.2 µg/L using the 90th percentile ingestion rate applied by EPA (Table 4). At the 99th percentile the calculated guideline value is 0.1–0.5 µg/L across all age groups. Thus, arguably, a guideline based on more commonly accepted uncertainty values would be 0.1 µg/L for the most sensitive groups.

### 5.3. Anatoxin-a and Anatoxin-a(S)

While anatoxin-a and anatoxin-a(S) act at the same neuronal synapse, their molecular targets and toxicity profiles are different. The LD_50_ for anatoxin-a via the oral route is 100 and 10-fold greater than that of anatoxin-a(S) and MCLR, respectively. Much uncertainty exists on the oral toxicity of anatoxin-a(S) given that few or no studies have been conducted on its toxicity via the oral route and in general toxicology studies of anatoxin-a(S) essentially ceased in the late 90’s due to a lack of material or available toxic strains. There is also no or limited data on its occurrence in the Great Lakes region.

Given the lack of repeat dose oral exposure studies it is impossible to establish a reference dose, NOAEL, or LOAEL at this time for anatoxin-a preventing the establishment of any kind of numerical limits for this toxin. A similar conclusion was recently reached by the USEPA [300]. The LD_50_ via oral gavage in mice is >10,000 µg/kg·b.w. and mice exposed to sub-lethal doses apparently recover without any lingering symptoms. Effects on reproductive tissue have been reported with repeat doses using IP injection, but not by the oral route making it difficult to establish a drinking water guideline for anatoxin-a using reproductive toxicity as an endpoint.

### 5.4. Saxitoxin

According to Munday and Reeve [318] currently no sub-acute repeat oral dosing studies of saxitoxin in animals have been reported using approved protocols. In contrast, the LD_50_ for saxitoxin via the oral route is well known at approximately 200 µg/kg·b.w. in the mouse (Wiberg and Stevenson) and ranges from 91 µg/kg·b.w. in pigeons to 800 µg/kg·b.w. in monkeys (Table 2 in [319]).

A LOAEL for saxitoxins of 1.5 µg/kg·b.w. has been reported by the European CONTAM on marine biotoxins based on epidemiological data [290]. Unlike other cyanotoxins, human poisonings with saxitoxins or PSPs have been well documented due to frequent human consumption of shellfish containing hazardous levels of saxitoxins [320,321,322]. CONTAM reviewed approximately 500 reports of human illness and death from eating shellfish contaminated with saxitoxins. In all cases concentrations of saxitoxins in the shellfish consumed were determined by the mouse bioassay. As such, the congeners of saxitoxins consumed are unknown and data are provided in mouse units. Mouse units were converted to mass per volume of saxitoxin equivalents by multiplying the mouse units by 0.18 µg saxitoxin equivalents per kg, which is widely used as conversion factor [323].

The use of case reports of human illness often requires a number of assumptions in order to relate dosage to the human health outcome. The reports of human intoxication events used by CONTAM required several such assumptions. For example, in many cases the weight of victims was unknown and assumed to be 60 kg if an adult or average age adjusted weight [324,325]. In one case, the amount of shellfish consumed was estimated based on the number of empty shells found after the meal [324]. In another case, the amount of toxin present in the shellfish consumed was estimated by interpolating concentration in shellfish collected the day before and after the poisoning event [325]. In addition, details of how the mouse bioassay was performed were often not available making it unclear if the assay was performed in agreement with the FDA approved method, 959.08 from the Association of Official Analytical Chemists.

These assumptions contribute uncertainty in the calculation of the LOAEL of 1.5 µg/kg·b.w. To account for this, an uncertainty factor of 10 could be used in calculating a guideline value for saxitoxins. On the other hand, since the LOAEL was determined based on human cases of illness an uncertainty multiplier for interspecies differences is not required. In addition, considering a population size of 500 human cases of saxitoxin related illness there is less uncertainty variation in toxicity based on intraspecies differences. 

**Figure 15:**
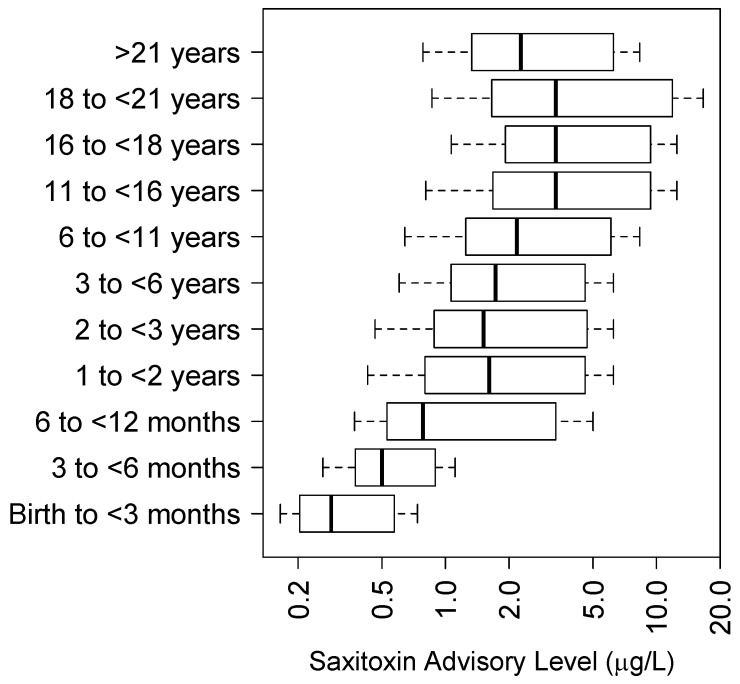
Variability in calculated saxitoxin advisory levels at all age levels and drinking water ingestion rates using a NOAEL of 0.5 µg/kg.

The CONTAM panel on saxitoxins divided the LOAEL by a factor of 3 to derive a NOAEL of 0.5 µg/kg·b.w. A guideline value can be calculated for saxitoxins using the same formula as for MCs, and this LOAEL of 0.5 µg/kg·b.w., as well as the DWI/BW ratio at the 90th percentile, and an uncertainty factor of 10. This produces a calculated guideline value for saxitoxins ranging from 0.2 to 1.5 µg/L across all age group categories (Figure 15) and 0.3 µg/L using the same ingestion rate applied by the EPA of 0.15 L/day for the first year of life (Table 4). On the other hand, for infants < 3 months old at the 99th percentile of the DWI/BW ratio, the most sensitive group, the guideline value is 0.16 µg/L. 

### 5.5. Issues and Considerations in Developing Numerical Limits for Cyanotoxins

The numerical limits discussed above are based on our current understanding of cyanotoxin toxicology. There are various aspects of cyanotoxins that are still relatively under studied, and thus not incorporated into the calculation of numerical limits. For example, for many cyanotoxins other endpoints have not been explored including epigenetic changes, developmental toxicity, behavioral, and cognitive deficits as well as reproductive effects in repeat oral dose studies. In addition, the contribution of cyanotoxins to the burden of chronic diseases in humans is difficult to assess, but has been explored in some epidemiological studies, particularly for MCs. These studies have not been considered in establishing numerical limits due to a lack of sufficient data. In particular, there are no diagnostic tests to assess cyanotoxin exposure in humans and as a result the exposure rate for most cyanotoxins in human populations is unknown. Finally, cyanotoxins rarely occur in isolation, but rather as a complex mixture. For example, many cyanobacteria genera are capable of producing multiple classes of toxins. Few or no studies have examined antagonistic or inhibitory effects of known cyanotoxin mixtures, although crude extracts containing unknown cyanotoxin mixtures have been used in animal studies. Given these uncertainties, the guideline values established as numerical limits are currently still provisionary.

A variety of issues are apparent with regards to the difficulty in establishing numerical limits for individual cyanotoxins. These include:

(1) There have been few repeat oral dose animal studies using purified cyanotoxin. These studies have traditionally served as the basis for developing numerical limits since ingestion is the primary route of cyanotoxin exposure.
(2) The contribution of cyanotoxins to chronic effects such as tumor promotion and cancer have not been considered in developing numerical limits for cyanotoxins, primarily due to a lack of data.
(3) Guideline values should be matched closely with monitoring capabilities. At present it is not clear if this is the case. For example, there is currently no known method that targets TTMCs.
(4) It is not clear whether the most sensitive individuals are protected at all levels of water ingestion rate and age group categories. In addition, other sensitive groups may not be protected such as those with underlying conditions that make them particularly sensitive to the effects of cyanotoxins.

## 6. Conclusions

Cyanobacteria are ancient organisms that have developed a number of adaptations that allow them to dominate nutrient rich lakes globally. CyanoHABs are a natural occurrence exacerbated by human activities including increased nutrient runoff, changes in land use, and possibly climate. In the Great Lakes region cyanoHABs most often occur in water bodies that maintain water temperatures above 20 °C for an appreciable period of time and that receive a large amount of nutrient input. The timing of cyanoHAB events at weekly or even monthly scales is difficult to predict and despite decades of research the ability to predict cyanotoxin concentrations and the exact environmental conditions under which cyanotoxin production occurs remains elusive. As such, regular monitoring for cyanoHABs and their toxins is necessary, as is experimental evidence indicating what maximal dosages ingested do not cause harmful effects. 

Cyanobacteria that occur in the Great Lakes region produce hundreds, or perhaps even thousands of toxic or otherwise bioactive substances. Among the most commonly reported are MCs, anatoxins, saxitoxins, and cylindrospermopsins as well as a variety of bioactive peptides. With the exception of cylindrospermopsin, the molecular mechanisms of toxicity and acute pathogenesis of these cyanotoxins are well known. For some cyanotoxins (i.e., MCs and cylindrospermopsins) the molecular mechanism of toxicity and/or pathological effects indicates they are possible carcinogens, and indeed, tumor promotion has been demonstrated in animal studies. However, for purposes of developing numerical limits on cyanotoxin exposure more repeat oral dosing studies are needed. In addition, the development of numerical limits may require the use of epidemiological data to account for the possible contribution of some cyanotoxins to chronic diseases.

MCs are clearly the most often detected, or targeted cyanotoxins in the Great Lakes region. However, historically there have been no regular monitoring programs for cyanotoxins in the Great Lakes and it is known that MCs co-occur with a variety of other toxic or otherwise bioactive peptides [326]. At present our understanding of the variability in cyanotoxin diversity across spatial and temporal scales in the Great Lakes region is relatively unknown, and the rate at which humans are exposed to these toxins has not been adequately addressed due to a lack of monitoring tools. Biomarkers of cyanotoxin exposure are needed in order to develop diagnostic tests and establish rates of human exposure to cyanotoxins. Such information will be useful in determining whether there are associations between cyanotoxin exposure and the development of chronic diseases.

In conclusion, cyanoHABs and their toxins are increasing across the Great Lakes region as a result of increased nutrient pollution of waterways and possibly climate change. In some waterways it is likely that nutrient inputs and the availability of internal nutrients (e.g., in sediments) cannot be reduced low enough to completely halt the development of cyanoHABs and toxin production anytime soon. For this reason long term strategies are needed for managing risk to human health from cyanotoxins. A thorough examination of these management strategies is beyond the scope of this report, but should include regular and improved monitoring of cyanotoxins in lakes or recreational environments and biota, reporting of such results to the public in a timely fashion, continued development of predictive models for forecasting cyanoHABs and their toxins, and improvements in drinking water treatment technologies or management of existing modern technologies to ensure cyanotoxins are efficiently removed without the production of toxic byproducts. Ultimately the problem of reducing cyanoHABs and their toxins in the Great Lakes will be addressed through intensive nutrient abatement programs in the watershed.

## Figures and Tables

**Table 1:** Mean and maximum MC concentrations (µg/L) at surface water locations in Lake Erie reported by Ohio EPA.

Site	Lake Erie Surface Water	Mean ^a^	Max ^a^	N ^b^
1	Lake Erie @ Gibralter Island Docks	3144.69	3144.69	1
2	Maumee Bay State Park Lake Erie Beach	21.38	570.00	52
3	Camp Perry Beach-Lake Erie	2.10	2.10	1
4	Lake Erie (Open Lake) East of Fairport Harbor	1.70	1.70	1
5	Lake Erie between Toledo/Oregon WTP Intakes	0.62	2.20	6
6	Lake Erie Ambient Site-Off Maumee Bay	0.58	3.20	30
7	Lake Erie Ambient Station-West Sister Island	0.31	2.80	34
8	Lake Erie Ambient Site-Port Clinton	0.28	2.00	33
9	Lake Erie North of Port Clinton	0.26	2.00	16
10	Lake Erie Off Detroit Near Canadian Border	0.19	2.00	22
11	Lake Erie @ Meinke Marina West	0.13	0.96	12
12	Lake Erie Ambient Station, Off Cedar Point	0.11	0.79	13
13	Lake Erie Ambient Station-Conneaut	0.10	0.49	5
14	Lake Erie Ambient Station-Fairport North	0.08	0.31	4
15	Lake Erie Ambient Site-Off Sandusky Bay	0.03	0.49	17
16	Lake Erie Ambient Station-Huron	0.02	0.58	30
17	Lake Erie Ambient Station-Rocky River	ND	ND	7
18	Lake Erie Ambient Station-Lorain West	ND	ND	7
19	Lake Erie Ambient Station-Wildwood	ND	ND	4
20	Lake Erie Fairport Transect Station 3	ND	ND	1
21	Lake Erie @ Channel Grove Marina	ND	ND	16
22	Lake Erie@Wild Wings Marina	ND	ND	10
23	Lake Erie@Lakefront Marina	ND	ND	3
24	Lake Erie@Brands Marina	ND	ND	4
25	Lake Erie Ambient Station-Geneva North	ND	ND	4

^a^ ND: not detected, ^b^ N: number of samples.

**Table 2:** Mean and maximum MC concentrations (µg/L) at drinking water intakes in Lake Erie reported by Ohio EPA.

Intake Site ^a^	Mean ^a^	Max ^a^	N ^b^
Put-In-Bay Water Treatment Plant Lake Erie Intake	5.83	340.00	63
Oregon Water Treatment Plant Lake Erie Intake	3.93	37.20	99
Camp Patmos Water Treatment Plant Lake Erie Intake	2.27	28.00	53
Carroll Water & Sewer Water Treatment Plant Lake Erie Intake	2.08	18.20	77
Toledo Water Treatment Plant Lake Erie Intake	1.24	50.00	1377
Painesville Water Treatment Plant Lake Erie Intake 2	1.10	3.90	9
Ottawa County Water Treatment Plant Lake Erie Intake	0.92	12.14	99
Lake Erie Utilities Water Treatment Plant Lake Erie Intake	0.69	4.33	54
Aqua Ohio-Mentor Water Treatment Plant Lake Erie Intake	0.67	2.20	8
Lake Co West Water Treatment Plant Lake Erie Intake	0.65	1.61	14
Kelleys Island Water Treatment Plant Lake Erie Intake	0.57	5.88	66
Fairport Harbor Water Treatment Plant Lake Erie Intake	0.39	1.20	7
Huron Water Treatment Plant Lake Erie Intake	0.34	4.62	31
Marblehead Water Treatment Plant Lake Erie Intake 1	0.33	3.80	61
Sandusky Water Treatment Plant Lake Erie Intake	0.30	2.50	73
Lake Co East Water Treatment Plant Lake Erie Intake	0.23	0.73	14
Avon Lake Water Treatment Plant Lake Erie 54 inch Intake	0.13	0.67	28
Lorain Water Treatment Plant Lake Erie Intake	0.12	0.61	24
Vermilion Water Treatment Plant Lake Erie Intake	ND	ND	13
Elyria Water Treatment Plant Lake Erie Intake	ND	ND	3
Painesville WTP Lake Erie Intake 1	ND	ND	1

^a^ ND = not detected, ^b^ N = number of samples.

**Table 3:** Mean and maximum MC concentrations (µg/L) in finished drinking water reported by Ohio EPA.

Plant	Mean	Max	N
Celina WTP	0.039	11	295
Carroll Water & Sewer WTP	0.113	3.56	77
Cadiz WTP	0.050	3.4	68
Toledo WTP	0.021	3.19	690
Kelleys Island WTP	0.025	1.68	67
Put-In-Bay WTP	0.016	0.6	60
Camp Patmos WTP	0.009	0.5	54
Campbell Soup Supply Co WTP	0.038	0.35	18
Oregon WTP	0.002	0.23	96

**Table 4:** Numerical limits for cyanotoxins in drinking water based on varying critical studies and toxic endpoints discussed in this report.

Toxin	Microcystins					Cylindrospermopsins			Saxitoxins
Source	^c^ WHO	^d^ EPA (U.S. and Canada)		This Report	This Report	EPA (U.S. and Canada)		This Report	This Report
Critical study	Fawell et al. 1999	Heinze 1999		Li et al. 2015	Chen et al. 2011	Humpage and Falconer 2002, 2003		Humpage and Falconer 2002, 2003	^e^ CONTAM
^a^ LOAEL/NOAEL (µg/kg/day)	40	50		5	1	30		30	0.5
End point	Liver Toxicity	Liver Toxicity		Central Nervous System Toxicity	Male Reproductive Toxicity	Kidney Toxicity		Kidney Toxicity	Peripheral Nervous System Toxicity
^b^ Age of Exposed	Adult	<6 years	>6 years	<6 years	<6 years	<6 years	>6 years	<6 years	<6 years
DWI/BW/day (L/kg/d)	0.03	0.15	0.03	0.15	0.15	0.15	0.15	0.15	0.15
Uncertainty Factor	1000	1000	1000	1000	1000	300	300	1000	3
Guideline Value (µg/kg)	0.96	0.3	1.6	0.03	0.01	0.7	3	0.2	0.3

^a^ LOAEL/NOAEL: lowest or no observed adverse effect level, ^b^ DWI and BW: Drinking Water Ingestion and Body Weight, ^c^ WHO: World Health Organization, ^d^ EPA: Environmental Protection Agency, ^e^ CONTAM: Panel on Contaminants in the Food Chain.
